# A systematic review of predictors and moderators of treatment response in psychological interventions for persisting forms of depression

**DOI:** 10.1111/bjc.12513

**Published:** 2024-12-31

**Authors:** Margaret Lyons, Jaime Delgadillo

**Affiliations:** ^1^ Clinical and Applied Psychology Unit, School of Psychology University of Sheffield Sheffield UK

**Keywords:** chronic, depression, moderators, predictors, psychosocial intervention, recurrent, treatment‐resistant

## Abstract

**Background:**

Although psychological interventions can be effective for the treatment of major depressive disorder, some patients' symptoms persist or rapidly recur after therapy. This study aimed to synthesize research findings on predictors and moderators of treatment response for persisting forms of depression, such as chronic, recurrent, and treatment‐resistant depression.

**Methods:**

A systematic review of studies investigating predictors and moderators of response to outpatient psychological treatment for adults with persisting forms of depression was conducted by searching Web of Science, Scopus, and PsycInfo. A total of 23 eligible studies were included, assessed for risk of bias, and summarized using a narrative synthesis.

**Results:**

Sixty‐five predictor and moderator variables were examined across studies, categorized into sociodemographic, clinical, interpersonal/personality, psychological, and treatment variables. Most variables were only examined in single studies, which were rarely adequately powered for predictor and moderator analyses. Among variables studied more frequently (age, gender, baseline depression severity, childhood trauma), only baseline depression severity was found to be a replicated and consistent predictor of poorer treatment response. Risk of bias was low to medium for the majority of studies.

**Limitations:**

Meta‐analysis could not be done due to methodological heterogeneity among studies.

**Conclusion:**

Our current understanding of significant predictors and moderators for persisting forms of depression is limited. A high level of baseline severity of depressive symptoms is so far the only variable consistently associated with poorer treatment response in this clinical population.

**Practitioner points**

- Among patients with persisting forms of depression, the only robust finding that emerges across available studies is that as baseline depression symptom severity increases, individuals are less likely to show a favourable response across treatments.
- Due to a lack of statistical power and inconsistent measurement of relevant variables in existing clinical trials for persisting forms of depression, understanding remains limited of what predicts poorer outcome across psychotherapies (prediction) and differential response to one psychotherapy versus another (moderation).
- At present, no individual variables reliably predict differential response to particular psychotherapies.

## INTRODUCTION

It is estimated that approximately 50% of individuals with major depressive disorder (MDD) will experience a chronic or recurrent course, and 20% will experience treatment‐resistant depression (TRD; Crown et al., 2002). Numerous terms are used within the literature to describe such persisting forms of depression, with three commonly discussed concepts being *chronic depression*, *treatment‐resistant depression*, and *recurrent depression*.

### Chronic depression

The occurrence of major depressive disorder symptoms for at least 2 years is commonly referred to as chronic depression, particularly for those who persistently meet criteria for clinically significant symptoms of MDD. In the current version of the Diagnostic and Statistical Manual of Mental Disorders (DSM‐V; American Psychiatric Association [APA], 2013), the concept of chronic depression is extended with the introduction of a persistent depressive disorder (PDD) category. Unlike in the DSM‐IV (APA, 2000), PDD has been introduced to include individuals who experience depressive symptoms over at least 2 years and not necessarily continuously but who do not meet MDD threshold (i.e., dysthymia). Chronic depression is associated with increased socio‐economic disadvantages and higher comorbidity with other psychiatric conditions when compared to single‐episode MDD (Murphy & Byrne, 2012).

### Treatment‐resistant depression (TRD)

Across the literature there is a lack of consensus on defining TRD. TRD is commonly defined as lack of response to two trials of antidepressant medication (ADM; Brown et al., 2019), although in some trials TRD is used to describe patients with no response to one treatment attempt (e.g., Wiles et al., 2013), and this definition has also been proposed to extend to include non‐response to prior psychological interventions (Gloster et al., 2020). Response to treatment can be defined in various ways, such as reduction of symptoms below a clinical threshold or clinically significant improvement. Alternative terms have been proposed to refer to TRD, such as ‘refractory depression’ or ‘difficult to treat depression’ (Demyttenaere & Van Duppen, 2019; McAllister‐Williams et al., 2020). In terms of frequency, a study in primary care by Thomas et al. (2013) found that 55% of their sample presented with TRD (defined as lack of response to at least 6 weeks of adequate dosage of ADM). TRD has a major personal and societal impact due to the cost of treatment, loss of work, increased suicidal risk, and caregiver burden (Demyttenaere & Van Duppen, 2019).

### Recurrent depression

The International Classification of Diseases (ICD‐10) defines recurrent depressive disorder as the experience of at least two recurring episodes of MDD (World Health Organization, 1993). Despite successful treatment for MDD, some studies suggest that 85% will experience a recurrence when followed‐up over 15 years (Hardeveld et al., 2009; Mueller et al., 1999). As with chronic depression and TRD, recurrent depression poses major personal and societal costs (Greden, 2001).

In the present review, we use the expression ‘persisting forms of depression’ to refer to all three concepts defined above and to emphasize the two central commonalities among these concepts: (1) the experience of symptoms of MDD and (2) the long‐term persistence or rapid recurrence of such symptoms after an initial episode of treatment. Such an approach has been taken in a review by McPherson and Senra (2022), who highlighted that there is much overlap between TRD, chronic depression, and recurrent depression. Approximately, 40% of patients with persisting forms of depression have treatment‐resistant symptoms (Schramm et al., 2020). Additionally, lower treatment compliance can be an issue in the management of persisting forms of depression, contributing to relapse in chronic and recurrent depression (Gopinath et al., 2007). Patients with persistent forms of depression often go through a series of treatments over a long period of time, following a trial‐and‐error process of searching for an adequate treatment option to alleviate symptoms and restore functioning. Therefore, a better understanding of which patient characteristics may be associated with better or poorer treatment response could help mental health services to improve the precision of diagnostic and treatment selection processes for patients with persisting forms of depression.

### Predictors and moderators of treatment response

Predictors and moderators are variables measured at (pre‐treatment) baseline assessments, which are statistically associated with treatment outcomes. Kraemer et al. (2002) explain that ‘moderators specify for whom and under what conditions treatment works’ (Kraemer et al., 2002, p. 878). This suggests that the interaction between a moderator variable and treatment predicts the outcome. As a result, moderators allow clinicians to make informed decisions on which treatment an individual patient is most likely to respond to. Predictors are defined as ‘a baseline measure that has a main effect on outcome but no interactive effect’ (Kraemer et al., 2002, p. 880). In clinical practice, this refers to variables that are generally associated with the treatment outcome, regardless of the treatment type. Hence, ‘predictors’ are general prognostic indicators, whereas ‘moderators’ are treatment‐specific variables.

Potential predictors and moderators of treatment outcomes have been extensively researched in the field of depression, for both pharmacological and psychological treatments (e.g., see Kessler et al., 2017; Papakostas & Fava, 2008; Tanguay‐Sela et al., 2022). However, reviews on predictors and moderators of treatment outcomes for persisting forms of depression are limited. For TRD specifically, predictors and moderators of treatment response have only been reviewed in relation to pharmacological treatment approaches (De Carlo et al., 2016). Some of the predictors of poorer response to pharmacological treatment included older age, higher numbers of past hospital admissions, presence of anxiety disorders, presence of personality disorders, and current suicidal risk (De Carlo et al., 2016). Similar reviews for chronic or recurrent depression were not found at the time of conducting this review.

### Aim

This systematic review aimed to identify predictors and moderators of change in psychological interventions offered to patients with persisting forms of depression, in order to determine which types of variables have been examined and which ones have replicated empirical support.

## METHOD

The Preferred Reporting Items for Systematic reviews and Meta‐Analyses (PRISMA) statement was used as a guideline for this review (Page et al., 2021). A review protocol was pre‐registered with PROSPERO in November 2022 (reference: CRD42022379257), prior to starting formal database searches.

### Search strategy

Three databases were systematically searched for relevant publications: Web of Science, PsycINFO, and Scopus. Studies published between database inception and search date (1 December 2022), which met pre‐defined inclusion and exclusion criteria, were sought. Additionally, search alerts informing the author of new publications added to databases were set up from 1 December 2022 until 13 April 2023. All newly added studies were screened for suitability. Studies in both the English and German languages were sought. Titles, abstracts, and indexes were searched across the databases using variations of the keywords ‘psychological intervention’, ‘chronic depression’, ‘recurrent depression’, and ‘treatment‐resistant depression’. For full search strategy, refer to the Supplementary Materials ‐ Table S1. The search strategy was informed by reviews on similar clinical populations and interventions (Ijaz et al., 2018; McPherson & Senra, 2022).

### Study eligibility

The Population, Intervention, Comparison, Outcomes and Setting (PICOS) framework was used to develop the research question and resulting inclusion and exclusion criteria (Table 1; Eriksen & Frandsen, 2018). Three depression subtypes were focused on, aimed at capturing the clinical population presenting with persisting depression symptomatology. TRD was defined as current MDD with at least one unsuccessful psychological or pharmacological intervention for depression. Due to the lack of consensus on defining TRD, an inclusive definition was chosen to allow review of a wider range of publications on TRD (Berlim & Turecki, 2007). Chronic depression was defined as the presence of depression symptoms, defined as scoring above clinical threshold on a validated screening measure or as identified via diagnostic interview, for at least 2 years or recurrence/relapse of depression symptoms during this time. This definition allows for inclusion of studies focusing on persistent depression as defined by the DSM‐V (APA, 2013). Recurrent depression was defined as an individual having a diagnosis of MDD at the time of beginning a psychological intervention, in addition to at least one past episode of depression (APA, 2013).

**TABLE 1:** Inclusion criteria.

	Inclusion criteria
Population	Patients (aged ≥18 years) with symptoms of major depressive disorder (in accordance with ICD‐10 or DSM‐V criteria or at the time of study valid diagnostic criteria) Diagnosis of major depressive disorder was identified via diagnostic interview or by scoring above clinical threshold on a validated screening measure In addition to the presence of MDD symptoms, patients meet the review criteria for treatment‐resistant, chronic, and/or recurrent depression
Intervention	Psychological intervention for persisting forms of depression. This includes any type of psychotherapeutic intervention but excludes combination treatments (psychotherapy and pharmacotherapy) and low‐intensity self‐help oriented interventions (e.g., brief guided self‐help, internet, or app‐enabled self‐help)
Comparator	Not applicable
Outcome	Standardized measure of depression symptoms (e.g., PHQ‐9, HRSD, BDI‐II), administered at least at baseline and post‐intervention Associations between at least one variable with post‐intervention depression symptoms is statistically analysed
Setting	Any treatment setting
Study design	Randomized controlled trials and cohort studies Written in English or German language

Abbreviations: BDI‐II, Beck Depression Inventory; DSM‐V, Diagnostic and Statistical Manual of Mental Disorders 5th Edition; HRSD, Hamilton Rating Scale for Depression; ICD‐10, International Classification of Diseases 10th revision; PHQ‐9, Patient Health Questionnaire‐9.

### Study selection

Search results from all three databases were combined. Following removal of duplicates, articles were screened by title and abstract. Subsequently, full‐text articles were retrieved and screened against the inclusion criteria. Additionally, backward and forward citation searches, as well as searching of relevant trials and reviews, were completed. A second reviewer was given a random selection of studies identified for full‐text screening (*n* = 10). There was no disagreement in study selection between the first author and second reviewer.

### Data extraction and synthesis

Data extraction and narrative synthesis were informed by published guidance (Boland et al., 2014) and relevant reviews (Amati et al., 2018; Ijaz et al., 2018; McPherson & Senra, 2022). Extraction of relevant data was completed by the first author and independently replicated by the second author. Predictors and moderators were defined in accordance with Kraemer et al. (2002), and this allowed for the inclusion of a wide and heterogeneous range of constructs and measures in primary studies as potential predictors and moderators. For secondary data analysis publications, the main trial publication was sought for data extraction where needed. Information was summarized in tables, with information from studies utilizing the same original dataset clustered together. Due to the range of examined predictor and moderator variables, these were categorized into relevant groups and a narrative synthesis was conducted. The present study specifically extracted data from psychotherapy studies or psychotherapy arms of clinical trials where psychotherapy was compared against control groups (data from control groups, including combination treatments, were not extracted).

### Risk of bias assessment

Risk of bias was assessed using the Critical Appraisal Skills Programme (CASP) case–control, cohort study, or randomized controlled trial checklists (CASP, 2022). CASP checklists were chosen for risk of bias assessment due to the simplicity in applying and appropriate checklists being available for each of the included study designs. For secondary data analysis publications, the main trial publication was sought to complete the risk of bias assessment.

The first author conducted risk of bias assessments for all studies. Furthermore, two additional independent reviewers also assessed all studies for risk of bias. Inter‐rater reliability, calculated and interpreted using the Kappa statistic (Cohen, 1960; Landis & Koch, 1977), was extremely high between the author and both additional raters, *κ* = .960 (SE = .020, *p* < .001). Disagreement in ratings was discussed, and a consensus was reached without the need of an additional reviewer.

## RESULTS

### Search results

**FIGURE 1:**
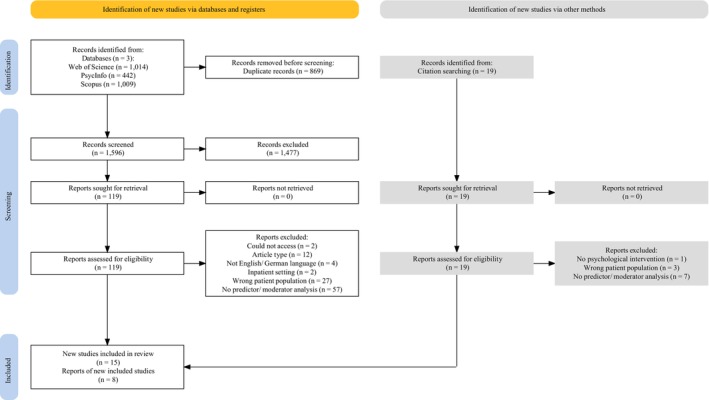
PRISMA diagram outlining study selection process.

The study selection process is outlined in Figure 1 using a PRISMA diagram (Page et al., 2021). Following the database searches, studies from all three databases were combined (*n =* 2465). Duplicates were removed (*n* = 869), and the remaining articles (*n* = 1596) were screened by title and abstract for relevance. For the remaining articles (*n* = 119), full‐text articles were retrieved and screened against the inclusion criteria. Database searches yielded 15 studies for the systematic review. Citation searches led to identification of a further eight studies. A total of 23 studies were included in the review.

### Study and participant characteristics

An overview of study and participant characteristics is shown in Table 2. Out of the included studies, the majority were randomized controlled trials (RCTs) (*n* = 20). The reviewed studies were conducted in the United States of America, the United Kingdom, and European countries. Sixteen studies were secondary data analyses to six other RCTs. The majority examined chronic depression (*n* = 16), with a further two studies looking at patients with co‐occurring chronic depression and TRD. Four studies looked at TRD only, and one study at recurrent depression. All studies assessed the presence of a depressive disorder utilizing, at the time of participant recruitment, widely accepted diagnostic criteria, namely the ICD‐10, DSM‐IV, and DSM‐V (APA, 2000, 2013; World Health Organization, 1993). Eighteen studies utilized additional measures of depressive symptoms at assessment, with the majority (*n* = 15) using the clinician‐administered Hamilton Depression Rating Scale (HDRS).

**TABLE 2:** Overview of study characteristics.

Study/Country	Study design	Target population/TRD definition	Assessment criteria	Intervention	Comparison condition(s)	*N* (Total/Analysed)	Age (years): *M* (SD)	Gender (% female)	Ethnicity (% white)	Past failed treatments	Main depression outcome
Cladder‐Micus et al. (2018) Netherlands	RCT	TRD (no response to ≥1 ADM + ≥1 psychological intervention)	DSM‐IV; IDS‐SR 21+	MBCT (group)	TAU	106/106	47.1 (10.25)	62%	NR	100%	IDS‐SR/Continuous
Eisendrath et al. (2016) USA	RCT	TRD (≥2 failed adequate ADM trials)	DSM‐IV; HDRS_17_ 14+	MBCT (group)	HEP	173/173	MBCT: 47.1 (13.46); HEP: 45.2 (11.19)	76%	80%	MBCT: *M =* 2.9 HEP: *M =* 3.06	QUIDS‐SR/% reduction
Lopez and Basco (2015) USA	Case Control Study	TRD (≥2 failed adequate ADM trials)	DSM‐IV; QUIDS‐SR 11+	CBT	TAU	166/166	43.1 (12.8)	84%	51%	100%	QIDS‐SR/Continuous
Potijk et al. (2020) Netherlands	Retrospective Chart Review	Chronic AND TRD (≥2 failed treatments including ≥1 ADM)	DSM‐IV	CBASP	None	54/54	Early‐onset cases: 50.8 (10.2); Late‐onset cases: 52.3 (8.2)	65%	NR	Psychotherapy: 98% AD: 93%; ECT: 11%; inpatient: 33%	IDS‐SR/Continuous
Renner and Berry (2011) Austria	RCT	Recurrent	ICD‐10	CBT (group)	Self‐Help Group/WL	66/34	42.7 (8.7)	100%	0%	NR	CES‐D/Continuous
Stangier et al. (2021) Germany	RCT	Chronic	DSM‐V	MBT (group) + CBT (individual)	WL	48/48	MBT (51.58 (11.26); WL (48.92 (11.39)	75%	NR	NR	QUIDS‐C/Response and remission
Taubner et al. (2011) Germany	Case Control Study	Chronic	DSM‐IV	LTPP	Healthy Controls	40/40	LTPP: 39.2 (12.7) Controls: 37.1 (11.6)	80%	NR	Psychotherapy and/or ADM: 80%	BDI‐II/Continuous
Secondary Data Analysis to Fonagy et al. (2015)											
Rost et al. (2019) UK	RCT	Chronic and TRD (≥2 failed adequate treatments including ≥1 ADM)	DSM‐IV; HRSD_17_ 14+; BDI‐II 21+	LTPP	TAU	129/120	44.0 (10.31)	63%	81%	LTPP: *M* = 3.5 (SD = 1.4) TAU: *M* = 3.9 (SD = 1.8)	HRSD_17_/Continuous
Secondary data analysis to Keller et al. (2000):											
Arnow et al. (2003) USA	RCT	Chronic	DSM‐IV; HRSD_24_ 20+	CBASP	ADM/ ADM + CBASP	681/347	44.9 (10)^a^	65%^a^	92%^a^	80% (Psychotherapy and/or ADM)	HRSD_24_/Continuous
Denton et al. (2010) USA						681/171	42.8 (9.0)^a^	64%^a^	91%^a^	80% (Psychotherapy and/or ADM)	IDS‐SR_30_/Remission
Kocsis, Leon, et al. (2009) USA	RCT	Chronic	DSM‐IV; HRSD_24_ 20+	CBASP	ADM/ ADM + CBASP	681/429	45 (NR)	65%^a^	92%^a^	ADM: 58%^a^	HRSD_24_/Continuous
Manber et al. (2008) USA						681/681	43.5 (10.7)	65%	91%	80% (Psychotherapy and/or ADM)	HRSD_24_/Remission
Nemeroff et al. (2003) USA						681/681	43.5 (10.7)	65%	91%	80% (Psychotherapy and/or ADM)	HRSD_24_/Remission
Secondary data analysis to Phase 2 of Kocsis, Gelenberg, et al. (2009):											
Arnow et al. (2013) USA	RCT	Chronic	DSM‐IV; HRSD_24_ 20+	CBASP	BSP	491/224	CBASP: 45.6(11.3)^a^; BSP: 47.4 (11.2)^a^	**CBASP:** 54%^a^ **BSP:** 53%^a^	**CBASP**: 93%^a^ **BSP**: 89%^a^	NR	HRSD_24_/Continuous
Shankman et al. (2013) USA					BSP/ADM	491/491	44.2 (1.2)	56%	88%	NR	HRSD_24_/Continuous
Steidtmann et al. (2012) USA					BSP/ADM	491/473	44.2 (1.2)	56%	88%	NR	HRSD_24_/Continuous
Secondary data analysis to Michalak et al. (2015):											
Probst et al. (2020) Germany	RCT	Chronic	DSM‐IV	MBCT (group)	CBASP (group)/TAU	106/68	MBCT: 48.09 (11.62)^a^; CBASP: 51.03, (10.60)^a^	62%^a^	NR	NR	HRSD_24_/Continuous
Secondary data analysis to Schramm et al. (2017) and Schramm et al. (2019):											
Assmann et al. (2018) Germany	RCT	Chronic	DSM‐IV; HRSD_24_ 20+	CBASP	SP	268/268	44.9 (11.8)	66%	NR	Psychotherapy: 57%; ADM: 55%; Combination: 20%	HRSD_24_/Continuous
Bausch et al. (2020) Germany						268/256	44.9 (11.8)	66%	NR	Psychotherapy: 57%; ADM: 55%; Combination: 20%	HRSD_24_ + IDS‐SR/Continuous
Erkens et al. (2018) Germany						268/247	44.9 (11.8)	66%	NR	Psychotherapy: 57%; ADM: 55%; Combination: 20%	HRSD_24_/Continuous
Klein et al. (2018) Germany						268/256	44.9 (11.8)	66%	NR	Psychotherapy: 57%; ADM: 55%; Combination: 20%	HRSD_24_/Continuous
Serbanescu et al. (2020) Germany	RCT	Chronic	DSM‐IV; HRSD_24_ 20+	CBASP	SP	268/268	44.9 (11.8)	66%	NR	Psychotherapy: 57%; ADM: 55%; Combination: 20%	HRSD_24_/% change
Secondary data analysis to Wiles et al. (2013)											
Button et al. (2015) UK	RCT	TRD (no response to ≥1 ADM taken for at least 6 weeks)	ICD‐10; BDI‐II 14+	CBT	TAU	469/469	CBT: 49.2 (11.9); TAU: 50 (11.5)	72%	99%	ADM: 80%	BDI‐II/Continuous

*Note*: Participant demographic and clinical characteristics of participants provided for total sample where available.

Abbreviations: ADM, antidepressant medication; BDI‐II, Becks Depression Inventory II; BSP, Brief Supportive Psychotherapy; CBASP, Cognitive‐Behavioural Analysis System of Psychotherapy; DSM‐IV, DSM‐V, Diagnostic and Statistical Manual of Mental Disorders 4th Edition or 5th Edition; HDRS_17_, HDRS_24_, Hamilton Depression Rating Scale 17‐item or 24‐item version; HEP, Health Enhancement Programme; ICD‐10, International Classification of Diseases 10th revision; ISD‐SR, The Inventory of Depressive Symptomatology Self‐Report; LTPP, long‐term psychoanalytic psychotherapy; MBCT, mindfulness‐based cognitive therapy; MBT, Metta‐based therapy; NR, not reported; QUIDS‐SR, QUIDS‐C, Quick Inventory of Depressive Symptomatology Self‐Report or Clinician‐Rated; RCT, randomized controlled trial; SP, supportive psychotherapy; TAU, treatment as usual; TRD, treatment‐resistant depression; WL, wait list.

^a^ Denotes that information refers to the analysed sample only. Continuous refers to the use of a numerical (scale) variable as the primary outcome.

The most frequent psychological intervention offered to participants was cognitive‐behavioural analysis system of psychotherapy (CBASP) (*n* = 14). The remaining interventions offered were CBT (*n* = 3), mindfulness‐based cognitive therapy (MBCT; *n* = 3), long‐term psychoanalytic psychotherapy (LTPP; *n* = 2), and MBCT combined with CBT (*n* = 1). Psychological interventions were primarily delivered on an individual basis (*n* = 18). Only one study did not have a control condition. The remaining studies had varying control conditions, namely ADM (*n* = 7), other psychological or psychosocial interventions (*n* = 11), combination treatments (*n* = 5), treatment‐as‐usual alone (*n* = 5), wait‐list control (*n* = 2), and healthy controls (*n* = 1). Ten studies had two control conditions, which included Renner and Berry (2011), Probst et al. (2020), five secondary data analyses to Keller et al. (2000), and two secondary data analyses to Kocsis, Gelenberg, et al. (2009). The third secondary data analysis to Kocsis, Gelenberg, et al. (2009) excluded the ADM control condition (Arnow et al., 2013). Overall, the psychological interventions entailed eight to 60 sessions over an 8‐to‐60‐week period. The use of ADM was allowed in all conditions or in the ADM‐only conditions for the majority of studies (*n* = 16). For further details on intervention characteristics, see Supplementary Materials. A variety of outcome measures were used to assess possible predictors and moderator variables, with the majority of studies (*n* = 15) utilizing clinician‐rated measures of depression.

Across all studies the majority of participants were females, and where ethnicity was reported, predominantly of White ethnicity. In studies that provided information on past failed treatments prior to the index episode, past failed interventions were reported for the majority of the sample indicating that a significant proportion of participants met the review's criteria of TRD.

### Risk of bias assessment

Out of all included RCTs, nine were considered to have a low, 10 a medium, and one study a high risk of bias. Across all studies participants and therapists were not blinded to the allocation condition. No study completed a power analysis specifically for the predictor or moderator analysis. Other common reasons for increased risk of bias included small sample sizes restricting generalizability of findings, insufficient information on results such as missing *p*‐values or partial reporting of results for all measures for each timepoint, and some differences in baseline characteristics of participants across the conditions.

For the two case–control studies, one was rated as low risk of bias (Lopez & Basco, 2015) and one as high risk of bias (Taubner et al., 2011). Taubner et al. (2011) utilized a healthy control group, affecting validity of findings. Potijk et al. (2020), a cohort study, was rated as having a medium risk of bias. This is due to lack of follow‐up and lack of clarity on treatment fidelity. All reviewed studies utilized valid and reliable measures to assess outcomes and examined predictor or moderator variables. Further detail on all risk of bias assessments is summarized in the Supplementary Materials.

### Narrative synthesis – Predictors and moderators

Across all reviewed studies, 65 different variables were analysed as potential predictors or moderators. An overview of examined variables, statistical analyses, and key findings is outlined in Tables 3 and 4. To aid interpretation of findings, variables were categorized as follows: sociodemographic characteristics, clinical characteristics, interpersonal and personality factors, psychological factors, and treatment factors. Clinical characteristics were divided into four further sub‐categories: depression characteristics, baseline clinical characteristics, comorbidities, and trauma factors.

**TABLE 3:** Summary of statistical analyses and key findings regarding predictors and moderators of treatment response.

Study *N* (Total/Analysed)	Predictor or moderator/Individual or pooled analysis	Statistical analysis	Variable (measure)	Key findings	Effect size (if significant)
Cladder‐Micus et al. (2018) 106/106	Moderator/Pooled	ANCOVA	Age^a^ Baseline depression score (IDS‐SR)^a^ Childhood trauma (CTQ)^a^ Duration current episode^a^ Gender Mindfulness (FFMQ)^a^ *N* previous episodes^a^ Rumination (RRS)^a^ Self‐compassion (SCS)^a^ Treatment resistance (DM‐TRD)^a^	NS NS NS NS NS NS NS Moderator, *F* (1, 84) = 5.44, *p* = .02 NS NS	– – – – – – – Not reported – –
Eisendrath et al. (2016) 173/173	Moderator and Predictor/Pooled	Multivariate Regression	Age at onset^a^ Baseline anxiety (STAI)^a^ Childhood trauma (CTQ)^a^ Current Stress (PSS)^a^ Disability status Duration current episode^a^ Education^a^ Ethnicity Medical illness Minority and socio‐economic status *N* previous episodes Personality disorder (SCID)	NS Predictor** Predictor** Predictor** NS NS NS NS NS NS NS Predictor**	– Not reported Not reported Not reported – – – – – – – Not reported
Lopez and Basco (2015) 166/166	Predictor/Pooled	Multilevel Regression	Age^a^ Gender Ethnicity Marital status Baseline depression score (QIDS‐SR)^a^ Past inpatient treatment Personality disorder Substance‐related disorder	Predictor, *b* = .00, SE = .00, *t* = 2.46, *p* = .014 Predictor, *b* = .05, SE = .02, *t* = −2.14, *p* = .033 NS NS Predictor, *b* = −.00, SE = .00, *t* = −2.11, *p* = .036 Predictor, *b* = −.03, SE = .02, *t* = −2.11, *p* = .046 NS NS	Not reported Not reported – – Not reported Not reported – –
Potijk et al. (2020) 54/54	Predictor/Individual	Independent *t*‐test, Chi‐Square test	Age at onset	Predictor, *p* = .010	Not reported
Renner and Berry (2011) 66/34	Predictor/Pooled	Linear Regression	Age^a^ Duration of stay in Austria^a^ Education^a^ Generation of migration Number of children^a^ Traumatic events experienced (LEC)^a^ Traumatic events witnessed (LEC)^a^	Predictor, *b = −.05*, SE = .02, *p = *.004 Predictor, *b* = .03, SE = .01, *p* = .034 NS NS NS Predictor, *b* = .09, SE = .03, *p* = .004 NS	Not reported Not reported – – – Not reported –
Stangier et al. (2021) 48/48	Moderator/Individual	MANOVA	Compassion to others (CLS)^a^	NS	–
Taubner et al. (2011) 40/40	Moderator/Individual	Multiple Hierarchical Regressions	Reflective functioning (RFS)^a^	NS	–
Secondary data analysis to Fonagy et al. (2015):					
Rost et al. (2019) 129/120	Moderator/Individual	Multilevel Regression Model	Personality features (AIDA)	Moderator, *b* = −.91, *SE* = .44, *p* = .038	Not reported
Secondary data analysis to Keller et al. (2000):					
Arnow et al. (2003) 681/347	Predictor/Pooled	Multiple Regression Model	Baseline depression scores (HRSD_24_)^a^ Gender Therapeutic reactance (TRS)^a^ ‘*Inner Directed’ Factor* *‘Defiant/Oppositional’ Factor*	Predictor, *B* = .20, *p* = .037 NS Predictor *b* = .22, *p* = .04 *b* = .27, *p* = .012	Not reported – Not reported Not reported
Denton et al. (2010) 681/171	Predictor/Individual	Logistic regression	Dyadic discord (MAS)	Predictor, *χ* ^2^ = 8.8, *df* = 1, *p* = .003	OR = 2.8, 95% CI = 1.4–5.5
Kocsis, Leon, et al. (2009) 681/429	Moderator/Individual	Logistic regression	Treatment preference	Moderator, *χ* ^2^ = 13.29, *df* = 6, *p* = .039	Not reported
Manber et al. (2008) 681/681	Predictor/Pooled	ROC analysis	Age^a^ Gender Ethnicity Marital Status Employment Status Baseline depression score (HRSD_24_)^a^ Baseline anxiety score (HAM‐A)^a^ Age at onset^a^ Duration current episode^a^ Childhood trauma (CTS) Attributional style (ASQ)^a^ Social functioning (SAS)^a^ Treatment group	NS NS NS NS NS Predictor, *p* < .01 Predictor, *p* < .01 NS NS NS NS NS Predictor, *χ* ^2^ = 19.7, *p* < .001	– – – – – Not reported Not reported – – – – – Not reported
Nemeroff et al. (2003) 681	Moderator/Individual	Linear regression and LOCF analysis	Childhood Trauma (CTS)	Moderator, OR = 2.322, 95% CI = 1.225–4.066	–
Secondary data analysis to Phase 2 of Kocsis, Gelenberg, et al. (2009)					
Arnow et al. (2013) 491/224	Predictor/Pooled	Linear Mixed Regression	Age Gender Global functioning (GAF)^a^	NS NS Predictor, *F*(1, 1218) = 84.85, *p* < .001	– – Not reported
Shankman et al. (2013) 491/491	Moderation/Individual	Linear Mixed Regression	Dysfunctional Attitudes (DAS)^a^	Moderator, *b* = −.0009, *t* (285) = − 3.19, *p <* .01	Not reported
Steidtmann et al. (2012) 491/473	Predictor/Individual	Linear Mixed Regression	Treatment Preference	NS	–
Secondary data analysis to Michalak et al. (2015):					
Probst et al. (2020) 106/68	Moderator/Pooled	Multilevel regression model	Interpersonal Problems (IIP‐32)^a^ *‘vindictive/self‐centred’ subscale* *‘non‐assertive’ subscale*	Moderator Estimate = 5.12 (*SE* = 1.71); *p* < .01 Estimate = −9.14 (SE = 2.84); *p* < .01	Not reported Not reported
Secondary data analysis to Schramm et al. (2017) and Schramm et al. (2019):					
Assmann et al. (2018) 268/268	Moderator/Individual	ANCOVA	Anxiety Disorder (SCID)	Moderator, *F*_1,256_ = 7.06, *p* = .01	Not reported
Bausch et al. (2020) 268/256	Moderator and Predictor/Individual	Linear Mixed Model	Childhood Trauma (CTQ)	NS	–
Erkens et al. (2018) 268/247	Moderator/Individual	ANCOVA	Personality Disorder (SCID)	NS	–
Klein et al. (2018) 268/256	Moderator and Predictor/Pooled	ANCOVA	Childhood Trauma (CTQ) *Emotional neglect subscale*	Moderator, *F*(1, 244) = 4.253, *p* = .040 Moderator, *F*(1, 244) = 6.866, *p* = .009 and Predictor, F(1, 244) = 8.565, *p* = .004	Not reported *d* = −.60
Serbanescu et al. (2020)^b^ 268/268	Moderator/Pooled	Multivariable and lasso regression and k‐fold cross‐validation	Gender Age^a^ Being Single Married/cohabiting Separated/divorced Widowed	NS NS NS NS M*, *r* = .34 (95% CI, .32; .36) M*, *r* = .34 (95% CI, .32; .36)	– – – – *d* ≥ .10 *d* ≥ .10
Serbanescu et al. (2020)^b^ 268/268	Moderator/Pooled	Multivariable and lasso regression and k‐fold cross‐ validation	Education Being employed Presence of ≥1 morbidity Chronic depression Double depression Recurrent depression Age at onset^a^ Duration current episode^a^ Baseline depression score (IDS‐SR)^a^ Baseline depression score (HRSD_24_)^a^ Suicidality (BSSI)^a^ History of ≥1 suicide attempt Baseline anxiety (GAD‐7)^a^ Baseline anxiety (BSI)^a^ Baseline phobic anxiety (BSI)^a^ Axis I disorder (SCID) Axis II disorder (SCID) Global functioning (GAF)^a^ Social functioning (SASS)^a^ Quality of life (QLDS)^a^ Interpersonal problems (IIP‐32)^a^ Childhood emotional abuse (CTQ) Childhood emotional neglect (CTQ) Childhood physical abuse (CTQ) Childhood physical neglect (CTQ) Childhood sexual abuse (CTQ) Past treatment type Past inpatient treatment Treatment preference	NS NS NS M*, *r* = .34 (95% CI, .32; .36) NS M*, *r* = .34 (95% CI, .32; .36) NS M*, *r* = .34 (95% CI, .32; .36) M*, *r* = .34 (95% CI, .32; .36) M*, *r* = .34 (95% CI, .32; .36) NS NS NS NS NS M*, *r* = .34 (95% CI, .32; .36) M*, *r* = .34 (95% CI, .32; .36) M*, *r* = .34 (95% CI, .32; .36) M*, *r* = .34 (95% CI, .32; .36) M*, *r* = .34 (95% CI, .32; .36) NS NS M*, *r* = .34 (95% CI, .32; .36) NS NS NS NS NS NS	– – – *d* ≥ .10 – *d* ≥ .10 – *d* ≥ .10 *d* ≥ .10 *d* ≥ .10 – – – – – *d* ≥ .10 *d* ≥ .10 *d* ≥ .10 *d* ≥ .10 *d* ≥ .10 – – *d* ≥ .10 – – – – – –
Secondary data analysis to Wiles et al. (2013):					
Button et al. (2015) 469/469	Moderators/Pooled	Random Effects Regression Model	Age^a^ Baseline anxiety (CIS‐R)^a^ Baseline depression score (CIS‐R)^a^ Baseline depression score (BDI‐II)^a^ Baseline PTSD score (PC‐PTSD)^a^ Current Stress (SRRS)^a^ Duration current episode^a^ Dysfunctional attitudes (DAS)^a^ Education Longstanding illness Marital status Meta‐cognitive awareness (MAQ)^a^ *N* previous episodes^a^ Neuroticism (BFI)^a^	Moderator, *b* = .24 (95% CI .44, .04), *p* = .02 NS NS Moderator, *b* = .20 (95% CI .00, .39), *p* = .05 NS NS NS NS NS NS NS NS NS NS	Not reported – – – Not reported – – – – – – – – – – –

*Note:* M* – Composite Moderator Score calculated using variables with an effect size of *d* ≥ .10 regardless of significant level.

Abbreviations: AIDA, Anaclitic‐Introjective‐Depression Assessment; ANCOVA, Analysis of covariance; ASQ, Attributional Style Questionnaire for Negative Events; BDI‐II, Beck Depression Inventory; BFI, ‘Big Five’ Inventory; BSI, Brief Symptom Inventory; BSSI, Beck Scale for Suicide Ideation; CIS‐R, Clinical Interview Schedule ‐Revised; CLS, Compassionate Love Scale; CTQ, Childhood Trauma Questionnaire; CTS, Childhood Trauma Scale; DAS, Dysfunctional Attitude Scale; DM‐TRD, Dutch Measure for Quantification of Treatment Resistance in Depression; FFMQ, Five Facets Mindfulness Questionnaire; GAD‐7, General Anxiety Disorder‐7; GAF, Global Assessment of Functioning Scale; HAM‐A, Hamilton Anxiety Scale; HRSD, Hamilton Rating Scale for Depression; IDS‐SR, Inventory of Depressive Symptomatology Self‐Report; IIP‐32, Inventory of Interpersonal Problems; LEC, Life Events Checklist from the Clinical Administered PTSD Scale; LOCF, Last observation carried forward; MANOVA, Multivariate analysis of covariance; MAQ, Meta‐cognitive Awareness Questionnaire; MAS, Marital Adjustment Scale; NS, Non‐Significant; PC‐PTSD, Primary Care PTSD Screening Tool; PSS, Perceived Stress Scale; QIDS‐SR, Quick Inventory of Depressive Symptomatology Self‐report; QLDS, Quality of Life In Depression Scale; RFS, Reflective Functioning Scale; ROC, Receiver operating characteristic; RRS, Ruminative Response Scale; SAS, Social Adjustment Scale; SASS, Social Adaptation Self‐evaluation Scale; SCID‐II, Structured Clinical Interview; SCS, Self‐Compassion Scale; SRRS, Social Readjustment Rating Scale; STAI, State–Trait Anxiety Inventory; TRS, Therapeutic Reactance Scale.

^a^ Variables that were operationalized as continuous, remaining operationalized as binary variables for analysis.

^b^ Serbanescu et al. (2020) pooled potential moderator variables into a composite moderator score, therefore same *p*‐value.

**Significance levels were unavailable.

**TABLE 4:** Overview of predictors and moderators by variable.

Variable	Study	Treatment conditions (intervention/control)	Predictor/moderator	Significance
Sociodemographic characteristics				
Age	Arnow et al. (2013) Button et al. (2015) Cladder‐Micus et al. (2018) Lopez and Basco (2015) Manber et al. (2008) Renner and Berry (2011) Serbanescu et al. (2020)	CBASP/BSP CBT/TAU MBCT/TAU CBT/TAU CBASP/ADM; ADM + CBASP CBT/Self‐help group; WL CBASP/SP	Predictor Moderator Moderator Predictor Predictor Predictor Moderator	NS S NS S NS S NS
Duration of stay in Austria	Renner and Berry (2011)	CBT/Self‐help group; WL	Predictor	S
Education Level	Button et al. (2015) Eisendrath et al. (2016) Renner and Berry (2011) Serbanescu et al. (2020)	CBT/TAU MBCT/HEP CBT/Self‐help group; WL CBASP/SP	Moderator Both Predictor Moderator	NS NS NS NS
Employment status	Manber et al. (2008) Serbanescu et al. (2020)	CBASP/ADM; ADM + CBASP CBASP/SP	Predictor Moderator	NS NS
Ethnicity	Eisendrath et al. (2016) Lopez and Basco (2015) Manber et al. (2008)	MBCT/HEP CBT/TAU CBASP/ADM; ADM + CBASP	Both Predictor Predictor	NS NS NS
Gender	Arnow et al. (2003) Arnow et al. (2013) Cladder‐Micus et al. (2018) Lopez and Basco (2015) Manber et al. (2008) Serbanescu et al. (2020)	CBASP/ADM; ADM + CBASP CBASP/BSP MBCT/TAU CBT/TAU CBASP/ADM; ADM + CBASP CBASP/SP	Predictor Predictor Moderator Predictor Predictor Moderator	NS NS NS S NS NS
Generation of migration	Renner and Berry (2011)	CBT/Self‐help group; WL	Predictor	NS
Marital status	Button et al. (2015) Lopez and Basco (2015) Manber et al. (2008) Serbanescu et al. (2020)	CBT/TAU CBT/TAU CBASP/ADM; ADM + CBASP CBASP/SP	Moderator Predictor Predictor Moderator	NS NS NS NS
Minority/Socio‐economic status	Eisendrath et al. (2016)	MBCT/HEP	Both	NS
Number of children	Renner and Berry (2011)	CBT/Self‐help group; WL	Predictor	NS
Clinical characteristics				
Baseline clinical characteristics				
Baseline Depression Severity	Arnow et al. (2003) Button et al. (2015) Manber et al. (2008) Lopez and Basco (2015) Serbanescu et al. (2020)	CBASP/ADM; ADM + CBASP CBT/TAU CBASP/ADM; ADM + CBASP CBT/TAU CBASP/SP	Predictor Moderator Predictor Predictor Moderator	S S S S S
Baseline Anxiety	Button et al. (2015) Eisendrath et al. (2016) Manber et al. (2008) Serbanescu et al. (2020)	CBT/TAU MBCT/HEP CBASP/ADM; ADM + CBASP CBASP/SP	Moderator Both Predictor Moderator	NS S NS NS
Quality of Life	Serbanescu et al. (2020)	CBASP/SP	Moderator	S
Social Functioning	Manber et al. (2008) Serbanescu et al. (2020)	CBASP/ADM; ADM + CBASP CBASP/SP	Predictor Moderator	NS S
Global Functioning	Arnow et al. (2013)	CBASP/BSP	Predictor	S
Stress levels	Button et al. (2015)	CBT/TAU	Moderator	NS
PTSD levels	Button et al. (2015)	CBT/TAU	Moderator	NS
Suicidality	Serbanescu et al. (2020)	CBASP/SP	Moderator	NS
Suicide attempts ≥1	Serbanescu et al. (2020)	CBASP/SP	Moderator	NS
Depression characteristics				
Chronic MDD	Serbanescu et al. (2020)	CBASP/SP	Moderator	S
Depression onset (Age)	Eisendrath et al. (2016) Manber et al. (2008) Serbanescu et al. (2020) Potijk et al. (2020)	MBCT/HEP CBASP/ADM; ADM + CBASP CBASP/SP	Both Predictor Moderator Predictor	NS NS NS S
Duration of episode	Eisendrath et al. (2016) Manber et al. (2008) Serbanescu et al. (2020)	MBCT/HEP CBASP/ADM; ADM + CBASP CBASP/SP	Both Predictor Moderator	NS NS S
Double depression	Serbanescu et al. (2020)	CBASP/SP	Moderator	NS
*N* Previous Episodes	Eisendrath et al. (2016) Button et al. (2015)	MBCT/HEP CBT/TAU	Both Moderator	NS NS
Recurrent depression	Serbanescu et al. (2020)	CBASP/SP	Moderator	S
Treatment resistance	Cladder‐Micus et al. (2018)	MBCT/TAU	Moderator	NS
Comorbidities				
At least one morbidity	Button et al. (2015) Serbanescu et al. (2020)	CBT/TAU CBASP/SP	Moderator Moderator	NS NS
Anxiety disorder	Assmann et al. (2018)	CBASP/SP	Moderator	S
Axis I comorbidity	Serbanescu et al. (2020)	CBASP/SP	Moderator	S
Axis II comorbidity	Serbanescu et al. (2020)	CBASP/SP	Moderator	S
Personality disorder	Lopez and Basco (2015) Eisendrath et al. (2016) Erkens et al. (2018)	CBT/TAU MBCT/HEP CBASP/SP	Predictor Both Moderator	NS S NS
Substance disorder	Lopez and Basco (2015)	CBT/TAU	Predictor	NS
Disability	Eisendrath et al. (2016)	MBCT/HEP	Both	NS
Medical illness	Eisendrath et al. (2016)	MBCT/HEP	Both	NS
Trauma factors				
Childhood Trauma	Bausch et al. (2020) Cladder‐Micus et al. (2018) Eisendrath et al. (2016) Klein et al. (2018) Manber et al. (2008) Nemeroff et al. (2003)	CBASP/SP MBCT/TAU MBCT/HEP CBASP/SP CBASP/ADM; ADM + CBASP CBASP/ADM; ADM + CBASP	Both Moderator Both Moderator Predictor Moderator	NS NS S S NS S
Emotional Abuse	Serbanescu et al. (2020)	CBASP/SP	Moderator	NS
Emotional Neglect	Serbanescu et al. (2020)	CBASP/SP	Moderator	S
Physical Abuse	Serbanescu et al. (2020)	CBASP/SP	Moderator	NS
Physical Neglect	Serbanescu et al. (2020)	CBASP/SP	Moderator	NS
Sexual Abuse	Serbanescu et al. (2020)	CBASP/SP	Moderator	NS
Traumatic events (experienced)	Renner and Berry (2011)	CBT/Self‐help group; WL	Predictor	S
Interpersonal and personality factors				
Dyadic discord	Denton et al. (2010)	CBASP/ADM; ADM + CBASP	Predictor	S
Interpersonal features	Rost et al. (2019)	LTPP/TAU	Moderator	S
Interpersonal problems	Probst et al. (2020) Serbanescu et al. (2020)	CBASP/SP	Moderator Moderator	S NS
Psychological factors				
Attributional style	Manber et al. (2008)	CBASP/ADM; ADM + CBASP	Predictor	NS
Compassion to Others	Stangier et al. (2021)	MBT + CBT/WL	Moderator	NS
Dysfunctional Attitudes	Button et al. (2015)	CBT/TAU	Moderator	NS
Meta‐cognitive Awareness	Button et al. (2015)	CBT/TAU	Moderator	NS
Mindfulness Skills	Cladder‐Micus et al. (2018)	MBCT/TAU	Moderator	NS
Neuroticism	Button et al. (2015)	CBT/TAU	Moderator	NS
Reflective Functioning	Taubner et al. (2011)	LTPP/Healthy Controls	Moderator	NS
Self‐Compassion	Cladder‐Micus et al. (2018)	MBCT/TAU	Moderator	NS
Rumination	Cladder‐Micus et al. (2018)	MBCT/TAU	Moderator	S
Treatment factors				
Therapeutic Reactance	Arnow et al. (2003)	CBASP/ADM; ADM + CBASP	Predictor	S
Treatment preference	Kocsis, Leon, et al. (2009) Serbanescu et al. (2020) Steidtmann et al. (2012)	CBASP/ADM; ADM + CBASP CBASP/SP BSP/ADM	Predictor Moderator Predictor	S NS NS
Past treatment type	Serbanescu et al. (2020)	CBASP/SP	Moderator	NS
Past inpatient treatment	Lopez and Basco (2015)	CBT/TAU	Predictor	S

Abbreviations: ADM, antidepressant medication; BSP, Brief Supportive Psychotherapy; CBASP, Cognitive‐Behavioural Analysis System of Psychotherapy; HEP, Health Enhancement Programme; LTPP, Long‐Term Psychoanalytic Psychotherapy; MBCT, Mindfulness‐based Cognitive Therapy; MBT, Metta‐based therapy; MDD, major depressive disorder; NS, non‐significant; SP, supportive psychotherapy; TAU, treatment as usual; WL, wait list.

Two studies utilized cross‐validation approaches in their analysis of potential predictors and moderators (Manber et al., 2008; Serbanescu et al., 2020). Manber et al. (2008) used receiver operating characteristic (ROC) curve analysis, where once a significant predictor and cut‐off point is identified the sample is divided into two subgroups and predictor testing is re‐started for each subgroup separately. Serbanescu et al. (2020) used cross‐validation methods to calculate a composite moderator M* which included all tested potential moderator variables with an effect size ≥.10.

#### Sociodemographic characteristics

Across studies, 12 sociodemographic variables were analysed. Age was examined in seven studies with mixed findings. Four studies did not find age to be a predictor or moderator of treatment outcome (Arnow et al., 2013; Cladder‐Micus et al., 2018; Manber et al., 2008; Serbanescu et al., 2020). Button et al. (2015) found age to be a significant moderator, noting that higher age was associated with better treatment outcomes in CBT as opposed to treatment as usual (TAU). Two studies found age to be a significant predictor. Lopez and Basco (2015) found younger participants to show faster response rates to CBT and TAU compared to older participants. Similar findings were reported by Renner and Berry (2011), who compared CBT to a structured self‐help group (SHG).

Five studies did not find gender to be a significant predictor or moderator (Arnow et al., 2003, 2013; Cladder‐Micus et al., 2018; Manber et al., 2008; Serbanescu et al., 2020). In contrast, Lopez and Basco (2015) found gender to be a predictor of improvement rate, with female participants improving at a faster rate and showing greater benefit from CBT than male participants.

Marital status was not a significant predictor or moderator (Button et al., 2015; Lopez & Basco, 2015; Manber et al., 2008; Serbanescu et al., 2020). However, Serbanescu et al. (2020) additionally analysed ‘being divorced/widowed’ and ‘being separated’ as separate variables, both of which met threshold for inclusion in the overall calculation of a composite moderator score. Level of education (Button et al., 2015; Eisendrath et al., 2016; Renner & Berry, 2011; Serbanescu et al., 2020), ethnicity (Eisendrath et al., 2016; Lopez & Basco, 2015; Manber et al., 2008), employment status (Manber et al., 2008; Serbanescu et al., 2020), and minority and socio‐economic status (Eisendrath et al., 2016) were not found to be predictors or moderators.

Renner and Berry (2011) studied treatment approaches for Turkish women with recurrent depression who migrated to Austria. Therefore, additional sociodemographic variables were tested as possible predictors: generation of migration, number of children, and duration of stay in Austria. Only duration of stay was a significant predictor, with greater number of years lived in Austria associated with better outcomes.

#### Clinical characteristics

##### Baseline clinical characteristics

Baseline depression scores were consistently found to be predictors or moderators across all studies which examined this variable (*n* = 5). Baseline depression was assessed using self‐report (Beck Depression Inventory; Inventory of Depressive Symptomatology self‐report; Quick Inventory of Depressive Symptomatology self‐report) or clinician‐rated outcome measures (HRSD, Clinical Interview Schedule ‐ Revised). Three studies found that lower baseline depression levels were associated with better post‐intervention outcomes (Arnow et al., 2003; Button et al., 2015; Lopez & Basco, 2015; Manber et al., 2008). In Manber et al. (2008), this was specific to those receiving CBASP. In Button et al. (2015), this was specific to the CBT sample and only when baseline depression was assessed using a self‐report measure, but not using when using the clinician‐rated measure. Serbanescu et al. (2020) found participants with higher baseline depression severity to benefit significantly more from CBASP than supportive psychotherapy (SP).

Baseline anxiety, as well as general and phobic anxiety, was not a significant predictor or moderator (Button et al., 2015; Manber et al., 2008; Serbanescu et al., 2020). However, Eisendrath et al. (2016) measured state and trait anxiety using the State–Trait Anxiety Inventory and found that state anxiety predicted smaller reductions in depression symptoms. Quality of life had a moderating effect, with higher baseline quality of life showing better outcomes with SP than CBASP (Serbanescu et al., 2020). Likewise, participants with higher baseline general and social functioning responded better to SP than CBASP (Serbanescu et al., 2020). Arnow et al. (2013) found baseline global functioning to be a significant predictor, with higher baseline scores associated with lower post‐intervention scores. Contrary to Serbanescu et al. (2020), Manber et al. (2008) did not find baseline social functioning to be a significant predictor or moderator. Eisendrath et al. (2016) examined impact of baseline stress levels, noting that higher scores predicted poorer outcomes. However, Button et al. (2015) assessed stress levels following adverse life events using the Social Readjustment Rating Scale and found a non‐significant effect on outcomes. Baseline post‐traumatic stress disorder levels (Button et al., 2015), baseline levels of suicidality, and history of suicide attempts (Serbanescu et al., 2020) were not significant predictors or moderators.

##### Depression characteristics

Seven different variables were examined. Serbanescu et al. (2020) explored depression types (chronic, double depression, and recurrent depression) as potential moderators. Only double depression did not meet threshold for inclusion in the composite moderator variable. Chronic depression was associated with better response to CBASP and recurrent depression with better treatment response to SP. Number of previous episodes (Button et al., 2015; Eisendrath et al., 2016) and level of treatment resistance (Cladder‐Micus et al., 2018) were not significant predictors or moderators. Age of depression onset was not found to be a significant predictor or moderator in three studies (Eisendrath et al., 2016; Manber et al., 2008; Serbanescu et al., 2020). In contrast, Potijk et al. (2020) found that patients with late‐onset chronic depression (i.e., onset after 21 years of age) had significantly higher remission rates than those with early‐onset chronic depression. However, this difference was not found when comparing pre‐ to post‐intervention score changes on the Inventory of Depressive Symptomatology self‐report measure (Potijk et al., 2020). Duration of episode did not predict or moderate outcomes in two studies (Eisendrath et al., 2016; Manber et al., 2008) but did meet threshold for the composite moderator variable in Serbanescu et al. (2020) where longer episode duration was associated with better outcomes to CBASP as opposed to SP.

##### Comorbidities

Some comorbidities were found to be significant predictors or moderators. Serbanescu et al. (2020) found that participants with an Axis I comorbidity benefitted more from CBASP, whereas those with an Axis II disorder benefitted more from SP. For the same sample, an analysis by Assmann et al. (2018) showed that those with an anxiety disorder responded significantly better to CBASP than SP. Personality disorder presence was not associated with treatment outcome in two studies (Erkens et al., 2018; Lopez & Basco, 2015), but Eisendrath et al. (2016) found that presence predicted significantly worse outcomes. Presence of at least one morbidity (Button et al., 2015; Serbanescu et al., 2020), a substance‐related disorder (Lopez & Basco, 2015), a disability (Eisendrath et al., 2016), or medical illness (Eisendrath et al., 2016) did not impact on outcomes.

##### Trauma factors

There was some evidence to suggest that the experience of trauma can affect outcomes. Renner and Berry (2011) examined impact of lifetime traumatic events, witnessed and experienced, with only latter being a significant predictor. The higher the number of traumatic events experienced, the greater the benefit from CBT or the SHG was. Childhood trauma was assessed using the Childhood Trauma Questionnaire (CTQ) or Childhood Trauma Scale (CTS) across seven studies. Three did not find a significant relationship with treatment outcome (Bausch et al., 2020; Cladder‐Micus et al., 2018; Manber et al., 2008). It is important to note that Bausch et al. (2020) compared baseline only with 1‐ and 2‐year follow‐up depression scores. Klein et al. (2018) analysed data from the same trial as Bausch et al. (2020), however only focused on pre‐ and immediate post‐intervention scores. They found that overall presence of childhood trauma and childhood emotional neglect were moderators of treatment outcome, noting that CBASP should be preferred to SP. Additionally, childhood emotional neglect was also a significant predictor, the presence of which was associated with worse outcomes. Significant findings were reported by a further three studies. Eisendrath et al. (2016) found that only the experience of emotional abuse or neglect was predictive of poorer outcomes. Serbanescu et al. (2020) found only the experience of emotional neglect to be a moderator, noting that CBASP would be the preferred treatment compared to SP. The study by Nemeroff et al. (2003) was the only study that used the CTS instead of the CTQ. Nemeroff et al. (2003) found childhood trauma, as well as the CTS sub‐categories of parental loss, physical abuse, and neglect, to be moderators of outcome. If childhood trauma was present, CBASP showed better outcomes than pharmacological treatment alone.

#### Interpersonal and personality factors

Overall, few studies assessed interpersonal and personality variables. The presence of relationship challenges, described as dyadic discord, was found to predict lower remission rates (Denton et al., 2010). Furthermore, interpersonal problems measured using the Inventory of Interpersonal Problems were moderators in Probst et al. (2020). Those scoring high on the ‘vindictive/self‐centred’ subscale benefitted more from CBASP, whereas those scoring high on the ‘non‐assertive’ subscale benefitted more from MBCT. However, moderating effects of interpersonal problems were not supported by Serbanescu et al. (2020). Rost et al. (2019) found that certain personality features assessed using the Anaclitic‐Introjective‐Depression assessment moderated treatment outcomes. Those with ‘self‐critical’ or ‘needy’ features benefitted more from LTPP than TAU.

#### Psychological factors

The majority of psychological factors examined were not found to predict or moderate outcomes. This included attributional style (Manber et al., 2008), compassion to others (Stangier et al., 2021), dysfunctional attitudes (Button et al., 2015), meta‐cognitive awareness (Button et al., 2015), mindfulness skills (Cladder‐Micus et al., 2018), neuroticism (Button et al., 2015), reflective functioning (Taubner et al., 2011), and self‐compassion (Cladder‐Micus et al., 2018). Only rumination was a moderator, with higher baseline rumination associated with larger decrease in depression symptoms in MBCT (Cladder‐Micus et al., 2018).

#### Treatment factors

Results provide limited evidence that treatment factors affect outcome. Therapeutic reactance was examined by Arnow et al. (2003) and was found to be a predictor of outcome for CBASP only. Those who had higher ‘inner directed’ or ‘defiant oppositional’ scores had higher depression symptom reduction. Treatment preference was not found to affect outcomes in two studies (Serbanescu et al., 2020; Steidtmann et al., 2012) but did in Kocsis, Leon, et al. (2009). Kocsis, Leon, et al. (2009) reported that those receiving their preferred treatment had higher rates of remission and partial response. In terms of past treatment types, Serbanescu et al. (2020) did not find these to be moderators. However, Lopez and Basco (2015) found that past inpatient treatment was predictive of quicker symptom improvement.

## DISCUSSION

This systematic review examined potential predictors and moderators of response to psychological treatment for persisting forms of depression. A total of 23 studies examining 65 variables across five domains (sociodemographic, clinical, interpersonal/personality, psychological, and treatment variables) were included. Over half (57%) of these variables were not found to be significant predictors or moderators. In some cases (25%), findings were mixed and inconclusive. Eighteen percent of variables were found to be significant predictors or moderators but were mostly examined for chronic depression only and in individual studies, thus lacking replicated evidence. However, some variables were studied more often than others (in at least five studies), namely age, gender, baseline depression severity, and the experience of childhood trauma. These sociodemographic and clinical factors are therefore more closely examined in the discussion. Findings are compared to the wider literature on MDD, which refers to research into all subtypes of MDD (including single‐episode MDD) across the lifespan.

### Contribution to the evidence base

#### Sociodemographic characteristics

Gender was not found to be a predictor or moderator in most studies that examined the variable, which is consistent with the wider literature on depression (Cuijpers et al., 2014; Nilsen et al., 2013). In contrast, findings for age as a potential predictor or moderator were mixed. The quality of studies that reported non‐significant findings ranged from low to medium and were all RCTs (Cladder‐Micus et al., 2018; Manber et al., 2008; Serbanescu et al., 2020). Studies that found younger age to be associated with more favourable treatment outcomes were varied in quality, with one case–control and one high risk of bias study included (Lopez & Basco, 2015; Renner & Berry, 2011). When compared with wider MDD research, findings on age as a predictor remain inconclusive (Cuijpers et al., 2020; Nilsen et al., 2013). When considering the quality of studies examining age in this review, as well as the wider literature, caution should be taken when viewing age as a potential predictor or moderator.

#### Baseline depression severity

Baseline depression severity was consistently found to be a predictor of treatment response, with lower baseline severity associated with better outcomes (Arnow et al., 2003; Button et al., 2015; Lopez & Basco, 2015; Manber et al., 2008). However, it should be noted that Arnow et al. (2003) and Manber et al. (2008) conducted secondary data analyses on the same trial data (Keller et al., 2000). Nevertheless, baseline depression severity is noted to be a robust predictor of psychological treatment response. Three reviews on depression note that lower baseline severity is predictive of better outcomes (Lorenzo‐Luaces et al., 2017; Nilsen et al., 2013; Tunvirachaisakul et al., 2018). It would be relevant to examine whether remission rates varied between low‐ and high‐severity cases, since those with severe symptoms can experience substantial reductions in symptom severity whilst remaining clinically depressed after treatment.

In terms of moderating effects, only Serbanescu et al. (2020) conducted a moderator analysis in this review. Higher baseline scores in chronic depression indicated CBASP as the favoured treatment over brief supportive psychotherapy. In contrast, a meta‐analysis by Weitz et al. (2015) compared CBT versus ADM treatments for depression and did not find baseline depression severity to be a moderator. However, it is important to note that Serbanescu et al. (2020) compared two psychological treatment approaches, CBASP and SP. One of these interventions, CBASP, was specifically developed for individuals with chronic depression and is found effective (Cuijpers et al., 2010; Ijaz et al., 2018; Schramm et al., 2017). It is possible that more severe cases of chronic depression benefitted more from a targeted intervention (CBASP) than from a non‐directive approach (SP).

Given the consistency in findings that baseline depression severity is associated with treatment outcomes, there is some preliminary evidence to support baseline depression severity as a potential predictor or moderator for persisting forms of depression. Confidence is further increased given the low to medium risks of bias across studies. Similarly, the wider literature on depression repeatedly notes baseline severity to be associated with outcomes.

#### Childhood trauma

Presence of childhood trauma was supported in some studies as a potential predictor or moderator, whereas not in others. All studies were RCTs with low to medium risk of bias, indicating acceptable study quality. However, several studies utilized the same original data sources, while showing different outcomes. Consistent with findings of this review, wider research into the impact of childhood trauma on depression treatment outcomes is mixed and inconclusive. Given that childhood trauma is a well‐recognized risk factor for the development of MDD, a recent meta‐analysis by the Childhood Trauma Meta‐Analysis Study Group (Childhood Trauma Meta‐Analysis Study Group, 2022) explored whether it was also associated with differential treatment outcomes. No significant differences in outcome were found between those with and without childhood trauma. This contrasts with Nanni et al. (2012) and Nelson et al. (2017) whose meta‐analyses found that the presence of childhood trauma was predictive of poor outcomes. In conclusion, it is unclear whether childhood trauma is a robust predictor or moderator of outcomes in persisting forms of depression.

#### Other baseline characteristics

Several baseline characteristics were not significant predictors or moderators. Several sociodemographic characteristics were repeatedly not found to be predictors or moderators, such as education level, ethnicity, and marital status. This is consistent with wider MDD research, where demographic characteristics are generally not found to be useful criteria for treatment allocation (Sharpley & Bitsika, 2011). Baseline anxiety severity was examined by several studies in this review but was mostly not found to be a predictor or moderator of treatment outcomes. This is in contrast to the wider MDD literature, where baseline anxiety has been found to be a predictor of outcome (Kiosses et al., 2011; Papakostas & Fava, 2008; Tunvirachaisakul et al., 2018). Additionally, the presence of comorbidities is often considered to be predictive of worse outcomes in MDD (Tanguay‐Sela et al., 2022; Tunvirachaisakul et al., 2018). However, this review showed there is insufficient evidence to support this to be the case for persisting forms of depression.

### Methodological considerations

Several methodological limitations of the reviewed studies should be considered. First, predictor and moderator analyses were mostly secondary data analyses. A major limitation is that most studies were not adequately powered for exploratory and secondary analyses, making interpretability uncertain and likely explaining the lack of replication observed across studies. Kraemer and Blasey (2016) recommend sample sizes of 200 to 500 participants when examining several predictors using multiple linear regression analysis. To ensure accurate and valid results for multilevel regression analysis, sample size requirements are even higher (Moineddin et al., 2007). Among reviewed studies, those based on secondary analyses of some RCTs (e.g., Keller et al., 2000; Kocsis, Gelenberg, et al., 2009; Schramm et al., 2017) appear sufficiently powered, with the exception of those conducting cross‐validation analyses (Manber et al., 2008; Serbanescu et al., 2020).

Due to lack of clarity around sufficient power to detect effects, the findings should be viewed as exploratory (e.g., Serbanescu et al., 2020). Not only significant but also non‐significant findings should be interpreted with caution. For example, ethnicity was not found to be a significant predictor or moderator. However, two of the three studies examining this variable (Eisendrath et al., 2016; Manber et al., 2008) had samples of mostly white participants and therefore would have been unlikely to detect any significant associations. It is recommended that examined variables are chosen carefully and are appropriate for the available sample (Kraemer & Blasey, 2016). For significant findings, future studies are needed to ascertain the robustness of identified predictors and moderators. Additionally, a variable may be a significant predictor of post‐intervention depression severity but may not be a predictor of remission, relapse, or dropout. These nuances of predictor and moderator research need to be considered when conducting analysis and interpreting findings (Steketee & Chambless, 1992).

### Limitations

It is important to note that most of the examined significant predictors and moderators were found for studies examining cognitive‐based therapies for chronic depression (particularly CBASP). This is likely to limit the generalizability of findings to other psychological interventions, as well as to TRD and recurrent depression.

Meta‐analysis can help increase validity and confidence in findings compared to narrative synthesis alone (Valentine et al., 2010). Additionally, meta‐analysis can help detect small effect sizes by combining data from several trials (Blundell, 2014). Although meta‐analysis can theoretically be completed with as few as two studies (Valentine et al., 2010), meta‐analytic analysis was not considered appropriate for this systematic review. For each of the variables considered for meta‐analysis (age, baseline depression severity, childhood trauma), several factors contributed to lack of suitability. This includes the following: differences in experimental and control conditions, some studies utilizing the same original data source, differences in how outcomes were measured (e.g., post‐intervention depression scores, depression improvement rates, % change in depression symptoms), and different approaches to analyses. Importantly, as shown in Table 3, most studies did not provide sufficient statistical information per variable of interest (e.g., effect size indices), other than whether or not they were statistically significant in the analysis.

This review was restricted to peer‐reviewed publications. Therefore, the reviewed literature may be subject to publication bias, given that significant findings are more likely to be published than non‐significant findings (Franco et al., 2014). Exclusion of grey literature also means that studies not yet published may have been missed (Pappas & Williams, 2011). To reduce language bias, attempts were made to consider publications in one additional non‐English language (German); however, this does not completely eliminate language bias. Only full articles were considered for this review, and not abstracts and conference proceedings, thereby increasing the risk of up‐to‐date evidence being missed. A further limitation is that some full‐text papers could not be obtained; therefore, potential data may have been missed. The risk of bias assessment was completed using the CASP checklist, given availability of an appropriate checklist for each included study design. However, CASP checklists were designed to support the assessment of evidence. Therefore, not all questions from the CASP checklist were relevant to this review (e.g., whether study results are applicable to the local context or would improve current provision of care). CASP checklists also do not allow for in‐depth assessment of methodological approaches to predictor and moderator analyses (e.g., suitability of chosen statistical analyses) and subsequently arising sources of bias.

### Implications for future research

In view of the considerable heterogeneity of variables studied as potential predictors and moderators, and the fact that most studies were not adequately powered to conduct the intended statistical analyses, some recommendations for future research are warranted. The development of a common framework for baseline data collection and digital phenotyping of clinical samples with depressive symptoms could support better replication of findings in this field. Such a standard battery of assessments and variables could be achieved through a Delphi study which is informed by literature reviews of potentially relevant domains of predictors, such as the present study and other similar efforts (e.g., see Kessler et al., 2017). Many moderator analyses were carried out using samples from clinical trials that were not adequately powered to permit such exploratory analyses, and which collected batteries of measures that are idiosyncratic to that particular study and sample (i.e., not common to other clinical trials). Our findings indicate that such a practice is unfruitful, and this also limits the potential utility of individual‐patient data (IPD) meta‐analyses to advance knowledge in this area. IPD meta‐analyses rely on harmonizing heterogeneous variable sets from clinical trials, often resulting in the analysis of a sparse and limited number of common predictors (e.g., see Cuijpers et al., 2022). In view of this, proponents of a move towards *precision mental health care* have advocated for the development of large‐scale prospective cohorts and experimental studies that are specifically designed to have sufficient statistical power to examine multiple predictors and/or moderator variables simultaneously, in order to develop multivariable clinical prediction models to support personalized treatment selection decisions (Deisenhofer et al., 2024; Delgadillo & Lutz, 2020; DeRubeis, 2019; Kessler & Luedtke, 2021).

## CONCLUSION

This review identified baseline depression severity as the only well‐replicated predictor of treatment response for persisting forms of depression. Current evidence suggests that patients with persistent and severe‐level symptoms may benefit more from intensive cognitive‐behavioural interventions (e.g., CBT, CBASP), relative to supportive/non‐directive therapies.

## AUTHOR CONTRIBUTIONS

**Margaret Lyons:** Conceptualization; methodology; data curation; writing – original draft; writing – review and editing; investigation; formal analysis. **Jaime Delgadillo:** Conceptualization; methodology; supervision; writing – review and editing.

## FUNDING INFORMATION

No financial support was received for the conduct and preparation of this review article.

## CONFLICT OF INTEREST STATEMENT

The authors declare no conflicts of interest.

## SYSTEMATIC REVIEW PRE‐REGISTRATION REFERENCE IN PROSPERO DATABASE

CRD42022379257.

## Supporting information


Tables S1–S7

## Data Availability

Data sharing not applicable to this article as no datasets were generated or analysed during the current study.
