# Supplementary material for: A systematic review of predictors and moderators of treatment response in psychological interventions for persisting forms of depression
Source: Br J Clin Psychol. 2024 Dec 31;64(3):623–56. doi: 10.1111/bjc.12513 (PMC12334989; doi:10.1111/bjc.12513)
Supplement: Supplementary file 1 — Tables S1–S7 [file BJC-64-623-s001.docx]

**Supplemental Materials**

**Table S1**

*Search Strategy*

| Construct | Search Term |
| --- | --- |
| Intervention | “psycho* therap*” OR “psycho* intervention” OR “cognitive behaviour* therap*” OR CBT or “cognitive therap*” OR counselling |
| Clinical Population | “treatment resistant depression” OR “chron* depress*” OR “recu* depress*” OR “relap* depress*” OR “persist* depress*” |

*Note.* Constructs were combined using the Boolean operators OR / AND. The Boolean operator * was used to include varying endings of the given search term.

**Table S2**

*Table of Ineligible Studies with Reasons for Exclusion*

| **Author(s)** | **Year** | **Title** | **Exclusion Criteria** |
| --- | --- | --- | --- |
| Aagaard et al. | 2017 | The efficacy of psychoeducation on recurrent depression: a randomized trial with a 2-year follow-up | no predictor/moderator analysis as per inclusion criteria |
| Abbass | 2006 | Intensive Short-Term Dynamic Psychotherapy of treatment-resistant depression: a pilot study | no predictor/moderator analysis as per inclusion criteria |
| Abel et al. | 2016 | Sudden Gains in Treatment Resistant Depression | no predictor/moderator analysis as per inclusion criteria |
| Abel et al. | 2013 | Cognitive-behavioral therapy improved response and remission at 6 and 12 months in treatment-resistant depression | wrong article type (commentary) |
| Andrews et al. | 2020 | Sudden Gains and Patterns of Symptom Change in Cognitive–Behavioral Therapy for Treatment-Resistant Depression | no predictor/moderator analysis as per inclusion criteria |
| Arnow et al. | 2007 | Dropouts versus completers among chronically depressed outpatients | no predictor/moderator analysis as per inclusion criteria |
| Aust et al. | 2022 | Efficacy of Augmentation of Cognitive Behavioral Therapy With Transcranial Direct Current Stimulation for Depression | participant criteria as per inclusion criteria not met |
| Barnhofer et al. | 2009 | Mindfulness-based cognitive therapy as a treatment for chronic depression: A preliminary study | participant criteria as per inclusion criteria not met |
| Bausch et al. | 2017 | Cognitive Behavioral Analysis System of Psychotherapy versus Escitalopram in Patients with Chronic Depression: Results from a Naturalistic Long-Term Follow-Up | wrong article type (letter to editor) |
| Bausch et al. | 2017 | The impact of childhood maltreatment on the differential efficacy of CBASP versus escitalopram in patients with chronic depression: A secondary analysis | participant criteria as per inclusion criteria not met |
| Beddig et al. | 2020 | Mindfulness-based focused attention training versus progressive muscle relaxation in remitted depressed patients: Effects on salivary cortisol and associations with subjective improvements in daily life | participant criteria as per inclusion criteria not met |
| Beutel et al. | 2022 | Recovery from chronic depression and structural change: 5-year outcomes after psychoanalytic and cognitive-behavioural long-term treatments (lac depression study) | participant criteria as per inclusion criteria not met |
| Blackburn & Moore | 1997 | Controlled acute and follow-up trial of cognitive therapy and pharmacotherapy in out-patients with recurrent depression | no predictor/moderator analysis as per inclusion criteria |
| Blalock et al. | 2008 | Cognitive and behavioral mediators of combined pharmacotherapy and psychotherapy of chronic depression. | no predictor/moderator analysis as per inclusion criteria |
| Bollman et al. | 2015 | Psychotherapy in old age: The Cognitive Behavioral Analysis System of Psychotherapy (CBASP) for chronically depressed elderly patients | inpatient setting |
| Bowie et al. | 2013 | Cognitive remediation for treatment-resistant depression: Effects on cognition and functioning and the role of online homework | no predictor/moderator analysis as per inclusion criteria |
| Chiesa et al. | 2015 | Mindfulness-based cognitive therapy vs. psychoeducation for patients with major depression who did not achieve remission following antidepressant treatment | no predictor/moderator analysis as per inclusion criteria |
| Cladder-Micus et al. | 2019 | Effects of mindfulness-based cognitive therapy on a behavioural measure of rumination in patients with chronic, treatment-resistant depression | no predictor/moderator analysis as per inclusion criteria |
| Conradi et al. | 2008 | Cognitive–behavioural therapy v. usual care in recurrent depression | participant criteria as per inclusion criteria not met |
| Constantino et al. | 2012 | The relation between changes in patients' interpersonal impact messages and outcome in treatment for chronic depression | no predictor/moderator analysis as per inclusion criteria |
| Constantino et al. | 2016 | Change in Patients' Interpersonal Impacts as a Mediator of the Alliance-Outcome Association in Treatment for Chronic Depression | no predictor/moderator analysis as per inclusion criteria |
| Corney et al. | 2005 | Thirty-six-month outcome data from a trial of counselling with chronically depressed patients in a general practice setting | participant criteria as per inclusion criteria not met |
| DeMello et al. | 2001 | A randomized controlled trial comparing moclobemide and moclobemide plus interpersonal psychotherapy in the treatment of dysthymic disorder | participant criteria as per inclusion criteria not met |
| Den Boer et al. | 2007 | Cognitive self-therapy for chronic depression and anxiety: A multi-centre randomized controlled study | participant criteria as per inclusion criteria not met |
| D'Urso et al. | 2013 | Transcranial Direct Current Stimulation and Cognitive-Behavioral Therapy: Evidence of a Synergistic Effect in Treatment-Resistant Depression | wrong article type (letter to editor/ case report) |
| Eisendrath et al. | 2008 | Mindfulness-based cognitive therapy for treatment-resistant depression: A pilot study | no predictor/moderator analysis as per inclusion criteria |
| Feldman et al. | 2014 | Mindfulness based cognitive therapy versus a health enhancement program for treatment resistant depression: a randomized controlled trial | wrong article type (study protocol) |
| Fonagy et al. | 2015 | Pragmatic randomized controlled trial of long-term psychoanalytic psychotherapy for treatment-resistant depression: The Tavistock Adult Depression Study (TADS) | no predictor/moderator analysis as per inclusion criteria |
| Forkmann et al. | 2016 | The Effects of Mindfulness-Based Cognitive Therapy and Cognitive Behavioral Analysis System of Psychotherapy added to Treatment as Usual on suicidal ideation in chronic depression: Results of a randomized-clinical trial | no predictor/moderator analysis as per inclusion criteria |
| Foroughi et al. | 2020 | The effectiveness of mindfulness-based cognitive therapy for reducing rumination and improving mindfulness and self-compassion in patients with treatment-resistant depression | no predictor/moderator analysis as per inclusion criteria |
| Frank et al. | 2007 | Randomized trial of weekly, twice-monthly, and monthly interpersonal psychotherapy as maintenance treatment for women with recurrent depression | no predictor/moderator analysis as per inclusion criteria |
| Friedman et al. | 2009 | Cognitive therapy augmentation versus CT switch treatment: A STAR*D report | no predictor/moderator analysis as per inclusion criteria |
| Furukawa et al. | 2018 | Cognitive-Behavioural Analysis System of Psychotherapy (CBASP), a drug, or their combination: differential therapeutics for persistent depressive disorder: a study protocol of an individual participant data network meta-analysis | participant criteria as per inclusion criteria not met |
| Graser et al. | 2006 | Effects of a 12-Week Mindfulness, Compassion, and Loving Kindness Program on Chronic Depression: A Pilot Within-Subjects Wait-List Controlled Trial | no predictor/moderator analysis as per inclusion criteria |
| Harley et al. | 2008 | Adaptation of dialectical behavior therapy skills training group for treatment-resistant depression | no predictor/moderator analysis as per inclusion criteria |
| Humer et al. | 2020 | Effects of alliance ruptures and repairs on outcomes | no predictor/moderator analysis as per inclusion criteria |
| Jarrett et al. | 2001 | Preventing recurrent depression using cognitive therapy with and without a continuation phase: A randomized clinical trial | participant criteria as per inclusion criteria not met |
| Keller et al. | 2000 | A comparison of nefazodone, the cognitive behavioral-analysis system of psychotherapy, and their combination for the treatment of chronic depression | no predictor/moderator analysis as per inclusion criteria |
| Khazanov et al. | 2020 | Distress and Anhedonia as Predictors of Depression Treatment Outcome: A Secondary Analysis of a Randomized Clinical Trial | treatment specification as per inclusion criteria not met (the psychotherapy condition was combined with ADM) |
| Klein et al. | 2003 | Therapeutic Alliance in Depression Treatment: Controlling for Prior Change and Patient Characteristics. | no predictor/moderator analysis as per inclusion criteria |
| Klein et al. | 2011 | Psychotherapy of chronic depression with cognitive behavioral analysis system of psychotherapy (CBASP) | wrong article type (overview of intervention) |
| Kocsis et al. | 2009 | Cognitive behavioral analysis system of psychotherapy and brief supportive psychotherapy for augmentation of antidepressant nonresponse in chronic depression: The REVAMP trial | no predictor/moderator analysis as per inclusion criteria |
| Kuyken et al. | 2008 | Mindfulness-Based Cognitive Therapy to Prevent Relapse in Recurrent Depression | participant criteria as per inclusion criteria not met |
| Kuyken et al. | 2015 | The effectiveness and cost-effectiveness of mindfulness-based cognitive therapy compared with maintenance antidepressant treatment in the prevention of depressive relapse/recurrence: Results of a randomised controlled trial (The PREVENT study) | participant criteria as per inclusion criteria not met |
| Lau | 2020 | Mindfulness-based cognitive therapy: A low intensity group program to prevent depressive relapse | wrong article type (book chapter) |
| Ledari et al. | 2018 | A Comparison Between the Effectiveness of Acceptance and Commitment Treatment and Behavioral Activation Treatment for Depression on Symptoms Severity and Rumination Among Patients with Treatment-Resistant Depression | no predictor/moderator analysis as per inclusion criteria |
| Leuzinger-Bohleber | 2017 | 'Consenting to be robbed so as not to be murdered': psychoanalytic treatments of chronically depressed patients in two parallel depression research studies | wrong article type (conference abstract) |
| Leuzinger-Bohleber et al. | 2019 | Outcome of psychoanalytic and cognitive-behavioural long-term therapy with chronically depressed patients: A controlled trial with preferential and randomized allocation | participant criteria as per inclusion criteria not met |
| Leuzinger-Bohleber et al. | 2020 | The LAC Study: A comparative outcome study of psychoanalytic and cognitive-behavioral long-term therapies of chronic depressive patients | wrong article type (book chapter) |
| Lo et al. | 2015 | Evaluating compassion–mindfulness therapy for recurrent anxiety and depression: A randomized control trial. | participant criteria as per inclusion criteria not met |
| Matsunaga et al. | 2010 | Psychosocial functioning in patients with treatment-resistant depression after group cognitive behavioral therapy | no predictor/moderator analysis as per inclusion criteria |
| McCullough et al. | 1997 | Cognitive-Behavior Therapy for Chronic Depression (CBT-CD): Combined national collaborative study | wrong article type (conference abstract) |
| McCullough | 2003 | Treatment for chronic depression using Cognitive Behavioral Analysis System of Psychotherapy (CBASP) | wrong article type |
| McLoughlin et al. | 2021 | Mindfulness based cognitive therapy for recurrent depressive disorder | wrong article type |
| Meister et al. | 2020 | Adverse events during a disorder-specific psychotherapy compared to a nonspecific psychotherapy in patients with chronic depression | participant criteria as per inclusion criteria not met |
| Melyani et al. | 2015 | Mindfulness based cognitive therapy versus cognitive behavioral therapy in cognitive reactivity and self-compassion in females with recurrent depression with residual symptoms | not in English or German language |
| Michalak et al. | 2016 | Mindfulness-Based Cognitive Therapy and a Group Version of the Cognitive Behavioral Analysis System of Psychotherapy for Chronic Depression: Follow-Up Data of a Randomized Controlled Trial and the Moderating Role of Childhood Adversities | wrong article type (letter to editor) |
| Michalak et al. | 2015 | A randomized controlled trial on the efficacy of mindfulness-based cognitive therapy and a group version of cognitive behavioral analysis system of psychotherapy for chronically depressed patients | no predictor/moderator analysis as per inclusion criteria |
| Minelli et al. | 2019 | Clinical efficacy of trauma-focused psychotherapies in treatment-resistant depression (TRD) in-patients: A randomized, controlled pilot-study | no predictor/moderator analysis as per inclusion criteria |
| Moeller et al. | 2020 | Rumination-focused cognitive behaviour therapy for non-responsive chronic depression: An uncontrolled group study | no predictor/moderator analysis as per inclusion criteria |
| Morriss et al. | 2016 | Efficacy and cost-effectiveness of a specialist depression service versus usual specialist mental health care to manage persistent depression: a randomised controlled trial | no predictor/moderator analysis as per inclusion criteria |
| Monnart et al. | 2019 | Treatment of Resistant Depression: A Pilot Study Assessing the Efficacy of a tDCS-Mindfulness Program Compared With a tDCS-Relaxation Program | no predictor/moderator analysis as per inclusion criteria |
| Morimoto et al. | 2014 | Neuroplasticity-based computerized cognitive remediation for treatment-resistant geriatric depression | no predictor/moderator analysis as per inclusion criteria |
| Murray et al. | 2010 | Relief of Chronic or Resistant Depression (Re-ChORD): A pragmatic, randomized, open-treatment trial of an integrative program intervention for chronic depression | no predictor/moderator analysis as per inclusion criteria |
| Nakagawa et al. | 2017 | Effectiveness of Supplementary Cognitive-Behavioral Therapy for Pharmacotherapy-Resistant Depression: A Randomized Controlled Trial | no predictor/moderator analysis as per inclusion criteria |
| Ninan et al. | 2002 | Symptomatic and syndromal anxiety in chronic forms of major depression: Effect of nefazodone, Cognitive Behavioral Analysis System of Psychotherapy, and their combination | no predictor/moderator analysis as per inclusion criteria |
| O'Mahen et al. | 2019 | Trajectories of Change in a Group Behavioral Activation Treatment for Severe, Recurrent Depression | participant criteria as per inclusion criteria not met |
| Ostacoli et al. | 2018 | Comparison of eye movement desensitization reprocessing and cognitive behavioral therapy as adjunctive treatments for recurrent depression: The European Depression EMDR Network (EDEN) randomized controlled trial | no predictor/moderator analysis as per inclusion criteria |
| Papageorgiou et al. | 2015 | Group Metacognitive Therapy for Severe Antidepressant and CBT Resistant Depression: A Baseline-Controlled Trial | no predictor/moderator analysis as per inclusion criteria |
| Renner et al. | 2018 | Exploring mechanisms of change in schema therapy for chronic depression | no predictor/moderator analysis as per inclusion criteria |
| Roehricht et al. | 2013 | An exploratory randomized controlled trial of body psychotherapy for patients with chronic depression | no predictor/moderator analysis as per inclusion criteria |
| Sayegh et al. | 2012 | Cognitive behavioural analysis system of psychotherapy for treatment-resistant depression: Adaptation to a group modality | no predictor/moderator analysis as per inclusion criteria |
| Schanche et al. | 2021 | Self-criticism and self-reassurance in individuals with recurrent depression: Effects of Mindfulness-Based Cognitive Therapy and relationship to relapse | participant criteria as per inclusion criteria not met |
| Schnitzler & Christenhusz | 2016 | Effectiveness of MBCT in addition to treatment as usual in a patient group with chronic anxiety or depression: A pilot-study | not in English or German language |
| Schramm et al. | 2015 | From animal behavior to human health an animal-assisted mindfulness intervention for recurrent depression | no predictor/moderator analysis as per inclusion criteria |
| Schramm et al. | 2017 | Effect of disorder-specific vs nonspecific psychotherapy for chronic depression: A randomized clinical trial | no predictor/moderator analysis as per inclusion criteria |
| Schuling et al. | 2018 | The Co-creation and Feasibility of a Compassion Training as a Follow-up to Mindfulness-Based Cognitive Therapy in Patients with Recurrent Depression | no predictor/moderator analysis as per inclusion criteria |
| Schuling et al. | 2020 | Recovery from recurrent depression: Randomized controlled trial of the efficacy of mindfulness-based compassionate living compared with treatment-as-usual on depressive symptoms and its consolidation at longer term follow-up | participant criteria as per inclusion criteria not met |
| Schearer et al. | 2019 | Refractory depression - cost-effectiveness of radically open dialectical behaviour therapy: findings of economic evaluation of RefraMED trial | no predictor/moderator analysis as per inclusion criteria |
| Scott | 1992 | Chronic Depression: Can Cognitive Therapy Succeed When Other Treatments Fail? | no predictor/moderator analysis as per inclusion criteria |
| Simpson et al. | 2003 | A randomized controlled trial to evaluate the effectiveness and cost-effectiveness of psychodynamic counselling for general practice patients with chronic depression | participant criteria as per inclusion criteria not met |
| Simpson et al. | 2000 | A randomised controlled trial to evaluate the effectiveness and cost-effectiveness of counselling patients with chronic depression | participant criteria as per inclusion criteria not met |
| Sledge | 1999 | Treatment of chronic depression with ICBT-CD: A preliminary treatment-outcome study assessing interpersonal and attributional correlates of chronic depression | could not access |
| Souza et al. | 2016 | Interpersonal psychotherapy as add-on for treatment-resistant depression: A pragmatic randomized controlled trial | participant criteria as per inclusion criteria not met |
| Stalsett et al. | 2012 | Existential dynamic therapy ("VITA") for treatment-resistant depression with Cluster C disorder: Matched comparison to treatment as usual | inpatient setting |
| Strauss et al. | 2012 | Group person-based cognitive therapy for chronic depression: A pilot randomized controlled trial | no predictor/moderator analysis as per inclusion criteria |
| Swan et al. | 2004 | Coping with depression: An open study of the efficacy of a group psychoeducational intervention in chronic, treatment-refractory depression | no predictor/moderator analysis as per inclusion criteria |
| Swan et al. | 2014 | Cognitive Behavioural Analysis System of Psychotherapy (CBASP) for chronic depression: Clinical characteristics and six month clinical outcomes in an open case series | no predictor/moderator analysis as per inclusion criteria |
| Ter Avest et al. | 2021 | Prospective associations between home practice and depressive symptoms in mindfulness-based cognitive therapy for recurrent depression: A 15 months follow-up study | participant criteria as per inclusion criteria not met |
| Ter Avest et al. | 2021 | Interplay between self-compassion and affect during Mindfulness-Based Compassionate Living for recurrent depression: An Autoregressive Latent Trajectory analysis | participant criteria as per inclusion criteria not met |
| Thase et al. | 1994 | Response to cognitive-behavioral therapy in chronic depression | could not access |
| Town et al. | 2017 | A randomised controlled trial of Intensive Short-Term Dynamic Psychotherapy for treatment resistant depression: the Halifax Depression Study | no predictor/moderator analysis as per inclusion criteria |
| Uebelacker et al. | 2012 | Adapted Behavior Therapy for Persistently Depressed Primary Care Patients An Open Trial | no predictor/moderator analysis as per inclusion criteria |
| Van Aalderen et al. | 2012 | The efficacy of mindfulness-based cognitive therapy in recurrent depressed patients with and without a current depressive episode: A randomized controlled trial | no predictor/moderator analysis as per inclusion criteria |
| Valenstein et al. | 2016 | Augmenting Ongoing Depression Care With a Mutual Peer Support Intervention Versus Self-Help Materials Alone: A Randomized Trial | Not psychological intervention |
| Van Aalderen et al. | 2015 | Long-term outcome of mindfulness-based cognitive therapy in recurrently depressed patients with and without a depressive episode at baseline | no predictor/moderator analysis as per inclusion criteria |
| Vittengl et al. | 2004 | Self-directed affiliation and autonomy across acute and continuation phase cognitive therapy for recurrent depression | no predictor/moderator analysis as per inclusion criteria |
| Vittengl et al. | 2010 | Improvement in social-interpersonal functioning after cognitive therapy for recurrent depression | no predictor/moderator analysis as per inclusion criteria |
| Vittengl et al. | 2009 | Deterioration in psychosocial functioning predicts relapse/recurrence after cognitive therapy for depression | no predictor/moderator analysis as per inclusion criteria |
| Vittengl et al. | 2019 | Estimating outcome probabilities from early symptom changes in cognitive therapy for recurrent depression | no predictor/moderator analysis as per inclusion criteria |
| Vittengl et al. | 2022 | Stability and Change in Relations Between Personality Traits and the Interpersonal Problems Circumplex During Cognitive Therapy for Recurrent Depression | no predictor/moderator analysis as per inclusion criteria |
| Vittengl et al. | 2022 | Does Symptom Linkage Density Predict Outcomes in Cognitive Therapy for Recurrent Depression? | no predictor/moderator analysis as per inclusion criteria |
| Watkins et al. | 2011 | Rumination-focused cognitive-behavioural therapy for residual depression: Phase II randomised controlled trial | participant criteria as per inclusion criteria not met |
| Watkins et al. | 2011 | An effectiveness trial of group cognitive behavioral therapy for patients with persistent depressive symptoms in substance abuse treatment | participant criteria as per inclusion criteria not met |
| Weck et al. | 2013 | The Relationship Between Therapist Competence and Homework Compliance in Maintenance Cognitive Therapy for Recurrent Depression: Secondary Analysis of a Randomized Trial | participant criteria as per inclusion criteria not met |
| Wells et al. | 2012 | Metacognitive therapy in treatment-resistant depression: A platform trial | no predictor/moderator analysis as per inclusion criteria |
| Werner et al. | 2018 | A cluster randomized controlled platform trial comparing group MEmory specificity training (MEST) to group psychoeducation and supportive counselling (PSC) in the treatment of recurrent depression | participant criteria as per inclusion criteria not met |
| Wiersma et al. | 2014 | The effectiveness of the cognitive behavioral analysis system of psychotherapy for chronic depression: A randomized controlled trial | no predictor/moderator analysis as per inclusion criteria |
| Wiles et al. | 2013 | Cognitive behavioural therapy as an adjunct to pharmacotherapy for primary care based patients with treatment resistant depression: Results of the CoBalT randomised controlled trial | no predictor/moderator analysis as per inclusion criteria |
| Wiles et al. | 2016 | Long-term effectiveness and cost-effectiveness of cognitive behavioural therapy as an adjunct to pharmacotherapy for treatment-resistant depression in primary care: Follow-up of the CoBalT randomised controlled trial | no predictor/moderator analysis as per inclusion criteria |
| Winnebeck et al. | 2017 | Brief training in mindfulness meditation reduces symptoms in patients with a chronic or recurrent lifetime history of depression: A randomized controlled study | no predictor/moderator analysis as per inclusion criteria |
| Wong | 2008 | Cognitive behavioral treatment groups for people with chronic depression in Hong Kong: A randomized wait-list control design | participant criteria as per inclusion criteria not met |
| Wong | 2009 | A six-month follow-up study of cognitive-behavioural treatment groups for Chinese people with depression in Hong Kong | participant criteria as per inclusion criteria not met |
| Yang et al. | 2005 | Effect of psychosocial intervention on quality of life and other factors in patients with recurrent depression | not in English or German language |
| Yasinski et al. | 2020 | Processes of change in cognitive behavioral therapy for treatment-resistant depression: Psychological flexibility, rumination, avoidance, and emotional processing | no predictor/moderator analysis as per inclusion criteria |
| Yeon-Hee & Byun | 2017 | Therapeutic Mechanism of MBCT and Clinical Application of MBCT(Mindfulness-Based Cognitive Therapy) Program on Chronic Depression | not in English or German language |

**Table S3**

*Overview of Intervention Characteristics for Eligible and Included Studies*

| **Study** | **Intervention** | **Comparison Condition(s)** | **Intervention Session *N /* Duration** | **Follow Up** | **ADM allowed** | **Intervention Efficacy** | | |
| --- | --- | --- | --- | --- | --- | --- | --- | --- |
| **Cladder-Micus et al. (2018**) | MBCT (group) | TAU | 8/8W | 3M, 6M | Yes | small to medium effect size (d = 0.35) for MBCT completers only | | |
| **Eisendrath et al. (2016)** | MBCT (group) | HEP + TAU | 8/8W | 24W, 36W, 52W | Yes | MBCT significantly % higher reduction in depression symptoms, no significant difference in remission rates | | |
| **Lopez and Basco (2015)** | CBT | TAU | 18/18W | No | Yes | CBT significant higher remission and clinically significant response rate | | |
| **Potijk et al. (2020)** | CBASP | None | ≥30/48W | No | Yes | medium effect size for CBASP at 12M (d = 0.51) | | |
| **Renner and Berry (2011)** | CBT (group) | Self-Help Group / WL | NR/16W | No | NR | Significant symptoms change on one of two outcome measures for CBT | | |
| **Stangier et al. (2021)** | MBT (group) + CBT (individual) | WL | 17/NR | 1M, 6M | Yes | High effect size for MBT+CBT | | |
| **Taubner et al. (2011)** | LTPP | Healthy Controls | NR/60W | No | No | High effect size for LTPP | | |
| Secondary Data Analysis to Fonagy et al. (2015) | | | | | | | |  |
| **Rost et al. (2019)** | LTPP | TAU | 60/60W | 24M, 30M, 48M | Yes | LTPP greater symptom reduction, *p* = .017 | | |
| Secondary Data Analysis to Keller et al. (2000) | | | | | | |  |  |
| **Arnow et al. (2003)** | CBASP | ADM /  ADM + CBASP | 16/12W | No | ADM conditions only | Combined treatment (CBASP+ADM) had significantly higher response rate | | |
| **Denton et al. (2010)** |  |  |  |  |  |  |  |  |
| **Kocsis et al. (2009a)** |  |  |  |  |  |  |  |  |

| **Manber et al. (2008)** | CBASP | ADM /  ADM + CBASP | 16/12W | No | ADM conditions only | Combined treatment (CBASP+ADM) had significantly higher response rate | |
| --- | --- | --- | --- | --- | --- | --- | --- |
| **Nemeroff et al. (2003)** |  |  |  |  |  |  |  |
| Secondary Data Analysis to Phase 2 of Koscis et al. (2009b) | | | | | | | |
| **Arnow et al. (2013)** | CBASP | BSP | 16-20/12W | No | Yes | Similar response rates across all three conditions, Significant symptom reduction across all conditions | |
| **Schankman et al. (2013)** |  | BSP / ADM |  |  |  |  |  |
| **Steidtmann et al. (2012)** |  | BSP / ADM |  |  |  |  |  |
| Secondary Data Analysis to Michalak et al. (2015) | | | | | | |  |
| **Probst et al. (2020)** | MBCT (group) | CBASP (group)/ TAU | NR/8W | 6M | Yes | No significant difference in remission rates between MBCT and CBASP, CBASP and MBCT more effective than TAU | |
| Secondary Data Analysis to Schramm et al. (2017) and Schramm et al. (2019) | | | | | | |  |
| **Assmann et al. (2018)** | CBASP | SP | 32/48W | No | No | CBASP significantly more effective than SP with small to medium effect size (d = 0.31) | |
| **Bausch et al. (2020)** |  |  |  | 12M, 24M |  |  |  |
| **Erkens et al. (2018)** |  |  |  | No |  |  |  |
| **Klein et al. (2018)** |  |  |  | No |  |  |  |
| **Serbanescu et al. (2020)** |  |  |  | No |  |  |  |
| Secondary Data Analysis to Wiles et al. (2013) | | | | | | |  |
| **Button et al. (2015)** | CBT | TAU | ≤ 18/NR | No | Yes | Significantly higher response rate for CBT | |

*Note.* Antidepressant medication (ADM), Brief Supportive Psychotherapy (BSP), Cognitive Behavioural Analysis System of Psychotherapy (CBASP), Health Enhancement Programme (HEP), Long Term Psychoanalytic Psychotherapy (LTPP), Months (M), Mindfulness-based Cognitive Therapy (MBCT), Metta-Based Therapy (MBT), Not reported (NT), Supportive Psychotherapy (SP), Treatment as usual (TAU), Weeks (W), Wait List (WL)

*Summary of Risk of Bias Assessments*

**Table S4**

*Overview Risk of Bias Assessment – Randomised Control Trials*

| **Study** | *Focused research question?* | *Appropriate Randomised?* | *Are all participants accounted for?* | *Participants and investigators blinded?* | *Are study groups similar?* | *Similar care across groups (except for experi- mental intervention)?* | *Effects of intervention reported comprehensively?* | *Precision of treatment effect reported?* | *Benefits greater than harm/ cost?* | *Results applicable to local population/ your context?* | *Experimental intervention greater value than existing interventions?* | ***Risk of Bias*** |
| --- | --- | --- | --- | --- | --- | --- | --- | --- | --- | --- | --- | --- |
| Cladder-Micus et al. (2018) | Y | Y | Y | N | Y | Y | P | Y | Y | Y | N | **MEDIUM** |
| Eisendrath et al (2016) | Y | Y | Y | N | Y | Y | P | N | CT | Y | CT | **MEDIUM** |
| Renner and Berry (2011) | Y | Y | Y | N | CT | CT | P | N | N | N | N | **HIGH** |
| Stangier et al. (2021) | Y | Y | Y | P | P | Y | Y | Y | Y | Y | CT | **MEDIUM** |
| Secondary data analysis to Fonagy et al. (2015) | | | | | | | | | | | | |
| Rost et al. (2019) | Y | Y | Y | P | P | Y | Y | Y | Y | Y | CT | **MEDIUM** |
| Secondary data analysis to Keller et al. (2000) | | | | | | | | | | | | |
| Arnow et al. (2003) | Y | Y | Y | P | Y | Y | P | Y | Y | Y | Y | **LOW** |
| Denton et al. (2010) | Y | Y | Y | P | Y | Y | P | Y | Y | Y | Y | **LOW** |
| Kocsis et al. (2009a) | Y | Y | Y | P | Y | Y | P | Y | Y | Y | Y | **LOW** |
| Manber et al. (2008) | Y | Y | Y | P | Y | Y | P | Y | Y | Y | Y | **LOW** |
| Nemeroff et al. (2003) | Y | Y | Y | P | Y | Y | P | Y | Y | Y | Y | **LOW** |
| Secondary Data Analysis to Kocsis et al. (2009b) | | | | | | | | | | | | |
| Arnow et al. (2013) | Y | Y | Y | P | Y | Y | P | Y | Y | Y | Y | **LOW** |
| Schankman et al. (2013) | Y | Y | Y | P | Y | Y | P | Y | Y | Y | Y | **LOW** |
| Steidtman et al. (2012) | Y | Y | Y | P | Y | Y | P | Y | Y | Y | Y | **LOW** |
| Secondary data analysis to Michalak et al. (2015) | | | | | | | | | | | | |
| Probst et al. (2020) | Y | Y | Y | N | Y | Y | Y | Y | Y | Y | CT | **LOW** |
| Secondary data analysis to Schramm et al. (2017) and Schramm et al. (2019) | | | | | | | | | | | | |
| Assmann et al. (2018) | Y | Y | Y | P | P | Y | Y | Y | CT | Y | Y | **MEDIUM** |
| Bausch et al. (2020) | Y | Y | Y | P | P | Y | Y | Y | CT | Y | Y | **MEDIUM** |
| Erkens et al. (2018) | Y | Y | Y | P | P | Y | Y | Y | CT | Y | Y | **MEDIUM** |
| Klein et al. (2018) | Y | Y | Y | P | P | Y | Y | Y | CT | Y | Y | **MEDIUM** |
| Serbanescu et al. (2020) | Y | Y | Y | P | P | Y | Y | Y | CT | Y | Y | **MEDIUM** |

| Secondary data analysis to Wiles et al. (2013) | | | | | | | | | | | | |
| --- | --- | --- | --- | --- | --- | --- | --- | --- | --- | --- | --- | --- |
| Button et al. (2015) | Y | Y | Y | P | CT | Y | Y | Y | Y | Y | N | **MEDIUM** |

*Note.* N – No (item not adequately addressed), Y – Yes (item adequately addressed), CT – Can’t tell if item adequately addressed, P – Partially (Item partially addressed).

**Table S5**

*Overview Risk of Bias Assessment – Case-Control Studies*

| **Study** | *Clearly focused research question?* | *Appropriate study method?* | *Cases recruited in an acceptable way?* | *Controls recruited in an acceptable way?* | *Was the exposure accurately measured to minimise bias?* | *Aside from experimental intervention, were groups treated equally?* | *Were confounding factors considered in design/ analysis?* | *Treatment effect size?* | *Precision estimate of treatment effect reported?* | *Do you believe the results?* | *Results applicable to local population/ your context?* | *Do results fit with other available evidence?* | **Risk of Bias** |
| --- | --- | --- | --- | --- | --- | --- | --- | --- | --- | --- | --- | --- | --- |
| Lopez and Basco (2015) | Y | Y | Y | Y | P | Y | Y | Medium to large effect size | Y | Y | Y | Y | **LOW** |
| Taubner et al. (2011) | Y | Y | Y | Y | P | N | CT | High | P | CT | N | CT | **HIGH** |

*Note.* N – No (item not adequately addressed), Y – Yes (item adequately addressed), CT – Can’t tell if item adequately addressed, P – Partially (Item partially addressed).

**Table S6**

*Overview Risk of Bias Assessment – Cohort Studies*

| **Study** | *Clearly focused research question?* | *Cohort recruited in an acceptable way?* | *Exposure accurately measured to minimise bias?* | *Outcome accurately measured to minimise bias?* | *All important confounding factors identified?* | *Were confounding factors considered in design/ analysis?* | *FU of subjects complete enough?* | *FU of subjects long enough?* | *Study results* | *Were results precise?* | *Do you believe the results?* | *Results applicable to local population/ your context?* | *Do results fit with other available evidence?* | *What are the implications of this study for practice?* | **Risk of Bias** |
| --- | --- | --- | --- | --- | --- | --- | --- | --- | --- | --- | --- | --- | --- | --- | --- |
| Potijk et al. (2020) | Y | Y | P | Y | Y | Y | Y | P | CBASP (group) more effective for late-onset than early-onset depression. | Y | Y | Y | CT | Limited due to lack of control group and sample size | **MEDIUM** |

*Note.* N – No (item not adequately addressed), Y – Yes (item adequately addressed), CT – Can’t tell if item adequately addressed, P – Partially (Item partially addressed).

**Table S7 - PRISMA 2020 Main Checklist**

*The Preferred Reporting Items for Systematic reviews and Meta-Analyses (PRISMA)2020 Item Checklist (Page et al., 2021)*

| **Topic** | **No.** | **Item** | **Location where item is reported** |
| --- | --- | --- | --- |
| **TITLE** |  |  |  |
| **Title** | 1 | Identify the report as a systematic review. | p.2, p. 7 |
| **ABSTRACT** |  |  |  |
| **Abstract** | 2 | See the PRISMA 2020 for Abstracts checklist |  |
| **INTRODUCTION** |  |  |  |
| **Rationale** | 3 | Describe the rationale for the review in the context of existing knowledge. | pp. 6-7 |
| **Objectives** | 4 | Provide an explicit statement of the objective(s) or question(s) the review addresses. | pp. 7 |
| **METHODS** |  |  |  |
| **Eligibility criteria** | 5 | Specify the inclusion and exclusion criteria for the review and how studies were grouped for the syntheses. | pp. 8-9 |
| **Information sources** | 6 | Specify all databases, registers, websites, organisations, reference lists and other sources searched or consulted to identify studies. Specify the date when each source was last searched or consulted. | p. 7 |
| **Search strategy** | 7 | Present the full search strategies for all databases, registers and websites, including any filters and limits used. | Supplemental Table 1 |
| **Selection process** | 8 | Specify the methods used to decide whether a study met the inclusion criteria of the review, including how many reviewers screened each record and each report retrieved, whether they worked independently, and if applicable, details of automation tools used in the process. | p. 8-10 |
| **Data collection process** | 9 | Specify the methods used to collect data from reports, including how many reviewers collected data from each report, whether they worked independently, any processes for obtaining or confirming data from study investigators, and if applicable, details of automation tools used in the process. | p. 10 |
| **Data items** | 10a | List and define all outcomes for which data were sought. Specify whether all results that were compatible with each outcome domain in each study were sought (e.g. for all measures, time points, analyses), and if not, the methods used to decide which results to collect. | p. 8-10 |
|  | 10b | List and define all other variables for which data were sought (e.g. participant and intervention characteristics, funding sources). Describe any assumptions made about any missing or unclear information. | p. 8-10 |
| **Study risk of bias assessment** | 11 | Specify the methods used to assess risk of bias in the included studies, including details of the tool(s) used, how many reviewers assessed each study and whether they worked independently, and if applicable, details of automation tools used in the process. | p. 10 |
| **Effect measures** | 12 | Specify for each outcome the effect measure(s) (e.g. risk ratio, mean difference) used in the synthesis or presentation of results. | not applicable |
| **Synthesis methods** | 13a | Describe the processes used to decide which studies were eligible for each synthesis (e.g. tabulating the study intervention characteristics and comparing against the planned groups for each synthesis (item 5)). | p. 10 |
|  | 13b | Describe any methods required to prepare the data for presentation or synthesis, such as handling of missing summary statistics, or data conversions. | not applicable |
|  | 13c | Describe any methods used to tabulate or visually display results of individual studies and syntheses. | p. 10 |
|  | 13d | Describe any methods used to synthesize results and provide a rationale for the choice(s). If meta-analysis was performed, describe the model(s), method(s) to identify the presence and extent of statistical heterogeneity, and software package(s) used. | p. 10 |
|  | 13e | Describe any methods used to explore possible causes of heterogeneity among study results (e.g. subgroup analysis, meta-regression). | not applicable |
|  | 13f | Describe any sensitivity analyses conducted to assess robustness of the synthesized results. | not applicable |
| **Reporting bias assessment** | 14 | Describe any methods used to assess risk of bias due to missing results in a synthesis (arising from reporting biases). | not applicable |
| **Certainty assessment** | 15 | Describe any methods used to assess certainty (or confidence) in the body of evidence for an outcome. | not applicable |
| **RESULTS** |  |  |  |
| **Study selection** | 16a | Describe the results of the search and selection process, from the number of records identified in the search to the number of studies included in the review, ideally using a flow diagram. | pp. 11-17 |
|  | 16b | Cite studies that might appear to meet the inclusion criteria, but which were excluded, and explain why they were excluded. | Supplemental Table 2 |
| **Study characteristics** | 17 | Cite each included study and present its characteristics. | pp. 13-17 |
| **Risk of bias in studies** | 18 | Present assessments of risk of bias for each included study. | Supplemental Tables S4-6 |
| **Results of individual studies** | 19 | For all outcomes, present, for each study: (a) summary statistics for each group (where appropriate) and (b) an effect estimate and its precision (e.g. confidence/credible interval), ideally using structured tables or plots. | Table 3 |
| **Results of syntheses** | 20a | For each synthesis, briefly summarise the characteristics and risk of bias among contributing studies. | p. 11-18 |
|  | 20b | Present results of all statistical syntheses conducted. If meta-analysis was done, present for each the summary estimate and its precision (e.g. confidence/credible interval) and measures of statistical heterogeneity. If comparing groups, describe the direction of the effect. | not applicable |
|  | 20c | Present results of all investigations of possible causes of heterogeneity among study results. | not applicable |
|  | 20d | Present results of all sensitivity analyses conducted to assess the robustness of the synthesized results. | not applicable |
| **Reporting biases** | 21 | Present assessments of risk of bias due to missing results (arising from reporting biases) for each synthesis assessed. | p. 18 |
| **Certainty of evidence** | 22 | Present assessments of certainty (or confidence) in the body of evidence for each outcome assessed. | Tables 3-4 |
| **DISCUSSION** |  |  |  |
| **Discussion** | 23a | Provide a general interpretation of the results in the context of other evidence. | pp. 36-40 |
|  | 23b | Discuss any limitations of the evidence included in the review. | pp. 41-42 |
|  | 23c | Discuss any limitations of the review processes used. | pp. 41-42 |
|  | 23d | Discuss implications of the results for practice, policy, and future research. | pp. 42 |
| **OTHER INFORMATION** |  |  |  |
| **Registration and protocol** | 24a | Provide registration information for the review, including register name and registration number, or state that the review was not registered. | p. 7 |
|  | 24b | Indicate where the review protocol can be accessed, or state that a protocol was not prepared. | p. 7 |
|  | 24c | Describe and explain any amendments to information provided at registration or in the protocol. | not applicable |
| **Support** | 25 | Describe sources of financial or non-financial support for the review, and the role of the funders or sponsors in the review. | not applicable |
| **Competing interests** | 26 | Declare any competing interests of review authors. | None. |
| **Availability of data, code and other materials** | 27 | Report which of the following are publicly available and where they can be found: template data collection forms; data extracted from included studies; data used for all analyses; analytic code; any other materials used in the review. | not applicable |

**Table S8 -** **PRISMA Abstract Checklist**

| **Topic** | **No.** | **Item** | **Reported?** |
| --- | --- | --- | --- |
| **TITLE** |  |  |  |
| **Title** | 1 | Identify the report as a systematic review. | Yes |
| **BACKGROUND** |  |  |  |
| **Objectives** | 2 | Provide an explicit statement of the main objective(s) or question(s) the review addresses. | Yes |
| **METHODS** |  |  |  |
| **Eligibility criteria** | 3 | Specify the inclusion and exclusion criteria for the review. | Yes |
| **Information sources** | 4 | Specify the information sources (e.g. databases, registers) used to identify studies and the date when each was last searched. | Yes |
| **Risk of bias** | 5 | Specify the methods used to assess risk of bias in the included studies. | Yes |
| **Synthesis of results** | 6 | Specify the methods used to present and synthesize results. | Yes |
| **RESULTS** |  |  |  |
| **Included studies** | 7 | Give the total number of included studies and participants and summarise relevant characteristics of studies. | Yes |
| **Synthesis of results** | 8 | Present results for main outcomes, preferably indicating the number of included studies and participants for each. If meta-analysis was done, report the summary estimate and confidence/credible interval. If comparing groups, indicate the direction of the effect (i.e. which group is favoured). | Yes |
| **DISCUSSION** |  |  |  |
| **Limitations of evidence** | 9 | Provide a brief summary of the limitations of the evidence included in the review (e.g. study risk of bias, inconsistency and imprecision). | Yes |
| **Interpretation** | 10 | Provide a general interpretation of the results and important implications. | Yes |
| **OTHER** |  |  |  |
| **Funding** | 11 | Specify the primary source of funding for the review. | No  (no funding to declare) |
| **Registration** | 12 | Provide the register name and registration number. | Yes |
